# Hepatic Expression of the Na^+^-Taurocholate Cotransporting Polypeptide Is Independent from Genetic Variation

**DOI:** 10.3390/ijms23137468

**Published:** 2022-07-05

**Authors:** Roman Tremmel, Anne T. Nies, Barbara A. C. van Eijck, Niklas Handin, Mathias Haag, Stefan Winter, Florian A. Büttner, Charlotte Kölz, Franziska Klein, Pascale Mazzola, Ute Hofmann, Kathrin Klein, Per Hoffmann, Markus M. Nöthen, Fabienne Z. Gaugaz, Per Artursson, Matthias Schwab, Elke Schaeffeler

**Affiliations:** 1 Dr. Margarete Fischer-Bosch Institute of Clinical Pharmacology, 70376 Stuttgart, Germany; roman.tremmel@ikp-stuttgart.de (R.T.); anne.nies@ikp-stuttgart.de (A.T.N.); B.vanEijck@gmx.de (B.A.C.v.E.); mathias.haag@ikp-stuttgart.de (M.H.); stefan.winter@ikp-stuttgart.de (S.W.); florian.buettner@ikp-stuttgart.de (F.A.B.); charlotte.koelz@ikp-stuttgart.de (C.K.); franziska_frisch@online.de (F.K.); pascale.mazzola@med.uni-tuebingen.de (P.M.); ute.hofmann@ikp-stuttgart.de (U.H.); kathrin.klein@ikp-stuttgart.de (K.K.); elke.schaeffeler@ikp-stuttgart.de (E.S.); 2 University of Tuebingen, 72076 Tuebingen, Germany; 3 iFIT Cluster of Excellence (EXC2180) “Image Guided and Functionally Instructed Tumor Therapies”, University of Tuebingen, 72076 Tuebingen, Germany; 4 Department of Pharmacy, Uppsala University, 75123 Uppsala, Sweden; niklas.handin@farmaci.uu.se (N.H.); fabienne.gaugaz@gmx.net (F.Z.G.); per.artursson@farmaci.uu.se (P.A.); 5 Institute of Human Genetics, University of Bonn, 53127 Bonn, Germany; p.hoffmann@uni-bonn.de (P.H.); markus.noethen@uni-bonn.de (M.M.N.); 6 Division of Medical Genetics, Department of Biomedicine, University of Basel, 4001 Basel, Switzerland; 7 Department of Genomics, Life & Brain Center, University of Bonn, 53127 Bonn, Germany; 8 Departments of Clinical Pharmacology, and of Pharmacy and Biochemistry, University of Tuebingen, 72076 Tuebingen, Germany

**Keywords:** *SLC10A1*, NTCP, genetic variants, epigenetics, metabolomics, HBV/HDV infection, remdesivir, DNA methylation, bulevirtide

## Abstract

The hepatic Na^+^-taurocholate cotransporting polypeptide NTCP/*SLC10A1* is important for the uptake of bile salts and selected drugs. Its inhibition results in increased systemic bile salt concentrations. NTCP is also the entry receptor for the hepatitis B/D virus. We investigated interindividual hepatic *SLC10A1*/NTCP expression using various omics technologies. *SLC10A1*/NTCP mRNA expression/protein abundance was quantified in well-characterized 143 human livers by real-time PCR and LC-MS/MS-based targeted proteomics. Genome-wide SNP arrays and *SLC10A1* next-generation sequencing were used for genomic analyses. *SLC10A1* DNA methylation was assessed through MALDI-TOF MS. Transcriptomics and untargeted metabolomics (UHPLC-Q-TOF-MS) were correlated to identify NTCP-related metabolic pathways. *SLC10A1* mRNA and NTCP protein levels varied 44-fold and 10.4-fold, respectively. Non-genetic factors (e.g., smoking, alcohol consumption) influenced significantly NTCP expression. Genetic variants in *SLC10A1* or other genes do not explain expression variability which was validated in livers (*n* = 50) from The Cancer Genome Atlas. The identified two missense *SLC10A1* variants did not impair transport function in transfectants. Specific CpG sites in *SLC10A1* as well as single metabolic alterations and pathways (e.g., peroxisomal and bile acid synthesis) were significantly associated with expression. Inter-individual variability of NTCP expression is multifactorial with the contribution of clinical factors, DNA methylation, transcriptional regulation as well as hepatic metabolism, but not genetic variation.

## 1. Introduction

The Na^+^-taurocholate cotransporting polypeptide NTCP (encoded by *SLC10A1*) is the main uptake transporter for conjugated bile salts in the human liver where it is exclusively expressed at the basolateral membrane of hepatocytes [1,2,3]. Together with its paralog, the apical bile salt transporter ASBT (*SLC10A2*) located in the apical membrane of ileal enterocytes [1], NTCP thereby plays an important role in the enterohepatic circulation of bile salts. Apart from its endogenous role as a bile acid transporter, NTCP mediates the transport of various drugs such as statins or the antifungal drug micafungin [2,3,4]. For instance, ~35% of hepatic rosuvastatin uptake depends on NTCP transport [5,6]. In addition, several compounds used to assess dynamic liver function in humans (e.g., bromosulfophthalein, indocyanine green) are substrates of NTCP [1]. Moreover, a number of drugs also inhibit NTCP in vitro thereby reducing the cellular uptake of bile salts (Table 1) indicating clinical consequences [1]. For instance, increased plasma bile salt concentrations have been reported in patients treated with cyclosporine A, ritonavir, saquinavir, and the novel highly potent NTCP inhibitor bulevirtide (formerly myrcludex B) [7,8,9,10], which recently received a conditional marketing authorization of the European Medicines Agency (EMA) and the U.S. Food and Drug Administration (FDA) for treatment of chronic hepatitis D virus (HDV) infection. Novel NTCP inhibitors have recently also been proposed as promising therapeutic options for the treatment of cholestatic liver disease [11].

Notably, in 2012, NTCP/*SLC10A1* has been identified as a cellular receptor for viral entry of the human hepatitis B virus (HBV) and its satellite hepatitis D virus (HDV) [41,42]. Susceptibility to HBV infection majorly depends on NTCP expression and overlaps with species and cell type-specific expression of NTCP [43]. NTCP mediates viral entry of HBV/HDV by its specific interaction with the pre-S1 domain of HBV large envelope protein, which is essential for infection of target cells. The amino acids 84–87 and 157–165 of NTCP have been elucidated to be important for HBV binding and infection [42]. While the crystal structures of bacterial homologs of the SLC10 family have been known for some time [44,45], the structure of mammalian NTCP has only recently been reported by three independent groups providing further insight into HBV recognition and entry [46,47,48].

Based on the various ways in which NTCP acts as an endogenous and pharmacological target as well as a potential susceptibility factor for HBV/HDV infection, it is clinically important to systematically elucidate the inter-individual variability in expression and function of NTCP. Subsequently, factors that may significantly explain inter-individual variability are important to enable in vivo prediction of NTCP function.

So far, several genetic variants in *SLC10A1* influencing transport activity of NTCP have been described predominantly in Asian or African populations [42,49]. Little is known about functional relevant *SLC10A1* variants in Caucasians [50], and particularly comprehensive genotype-phenotype studies are missing. NTCP deficient patients have been also identified. One patient carrying the missense variant p.R252H (rs147226818) [51] had a mild clinical phenotype, but elevated plasma bile salts. Another patient homozygous for p.S267F (rs2296651) presented with mild jaundice but developed no further clinical signs apart from hypercholanemia [52]. In addition, NTCP deficiency in monozygotic twins due to the p.S267F variant caused transient cholestasis, as well as persistent hypercholanemia [53]. As shown by Yan et al. [54], polymorphisms that alter NTCP transport function are also critical for viral entry, indicating that molecular mechanisms important for HBV/HDV entry overlap with those for bile salts uptake. Of note, the p.S267F variant has been shown to be protective against HBV/HDV infection and was associated with a reduced risk of developing advanced liver cancer [55].

Independent from the genome there is increasing evidence that regulatory, epigenetic, and metabolic factors may contribute to inter-individual variability of the expression of hepatic transporter proteins as recently shown for organic anion transporting polypeptides and organic cation transporters [56,57]. Therefore, in addition to descriptive and clinical factors, we comprehensively investigated genomic, epigenetic, and metabolic features to better explain interindividual variability of hepatic NTCP expression by the use of well-characterized human liver samples of Caucasian ancestry.

## 2. Results

### 2.1. Expression of SLC10A1/NTCP in Human Liver and Correlation with Clinical Data

**Figure 1:**
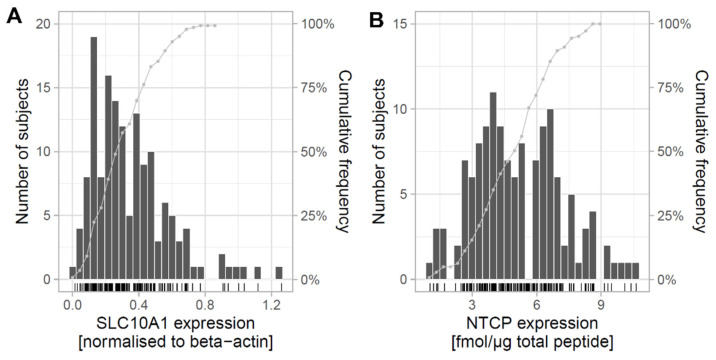

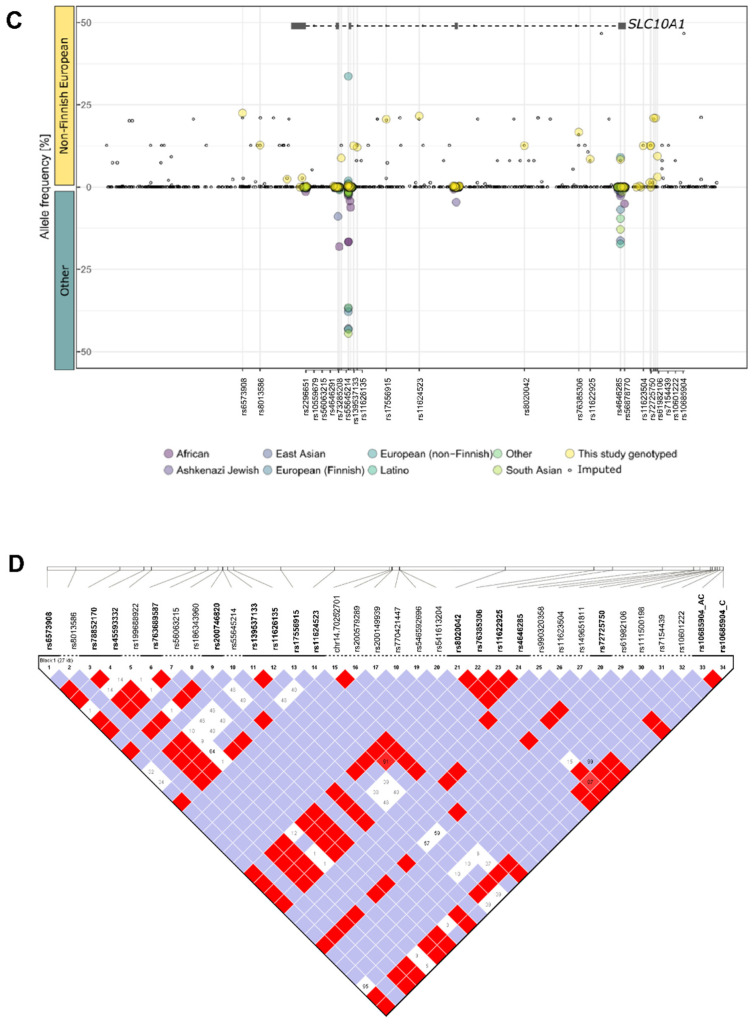
Expression and genetic variability of *SLC10A1*/NTCP in human liver. (**A**) Hepatic *SLC10A1* mRNA expression in 143 individuals. Results are presented as histogram, including cumulative frequencies. mRNA levels were measured using qPCR. (**B**) Hepatic NTCP protein levels in 143 individuals, which was analyzed by targeted proteomics using mass spectrometry. Results are presented as histogram, including cumulative frequencies. (**C**) Schematic overview of the *SLC10A1* locus (hg19: chr.14:70242135-70264007) with all analyzed (genotyped as well as imputed) SNPs in the 143 liver samples in relation to Genome Aggregation Database (gnomAD) data (gnomAD_v2.1_ENSG00000100652_2019_03_+UTR; data accessed in March 2019). On the top panel minor allele frequencies (MAF) greater than zero of variants detected in samples with European ancestry are shown. On the lower panel MAFs available in samples with other ethical backgrounds are shown on a reverse y-scale. Common variants (MAF > 5%) are highlighted using vertical gray lines and rs numbers. While yellow points are showing variants genotyped with NGS, MALDI-TOF MS, or Sanger sequencing, small black points are illustrating SNPs that have been imputed from global SNP array information on the same liver subjects (Infinium Global Screening Array GSA v2.0). (**D**) Pairwise linkage disequilibrium map of the *SLC10A1* gene region. The plot is created using Haploview and D’ values are shown.

In the present study, we first performed a detailed analysis of the hepatic expression of NTCP on mRNA and protein levels in 143 liver tissues of Caucasian origin. *SLC10A1* expression on mRNA level was measured using quantitative real-time PCR (qPCR), while the NTCP protein levels were determined using LC–MS/MS-based targeted proteomics. The *SLC10A1* mRNA expression showed a 44-fold variation (coefficient of variation: 66%) (Figure 1A) and was significantly affected by age, inflammation (i.e., C-reactive protein [CRP] levels), cholestasis, smoking, and alcohol consumption (Supplementary Table S1). At protein level, the expression of NTCP varied 10.4-fold (coefficient of variation = 38%; Figure 1B) and was also significantly associated with inflammation, smoking, and alcohol consumption (Supplementary Table S1). Protein and mRNA expression levels were significantly correlated (r_S_ = 0.44; *p* = 6 × 10^−8^; Supplementary Figure S1). 

### 2.2. Genetic Variability of SLC10A1 in Human Liver

In order to investigate the influence of genetic variation on *SLC10A1*/NTCP expression, we analyzed variants in the *SLC10A1* locus including the 2kb promoter region, all exonic and flanking intronic regions, as well as the 5′ promotor and 3′ untranslated region (UTR) for all 143 liver samples. Frequency distribution of all variants that have been investigated through genotyping (i.e., matrix-assisted laser desorption ionization time-of-flight mass spectrometry [MALDI-TOF MS], microarray technologies) and/or sequencing (i.e., Sanger sequencing, next-generation sequencing [NGS]) are listed in Supplementary Table S2. A total number of 34 variants were detected with common minor allele frequencies (MAF ≥ 5%; *n* = 17), rare frequencies (MAF < 5%; *n* = 5), or only in single individuals (*n* = 12). The genotype distribution of identified variants fitted to the Hardy-Weinberg equilibrium (Supplementary Table S2). Our results are in line with data of the large Exome-Genome-NGS-cohort of Non-Finnish Europeans (*n* = 56,000) from the genome aggregation database (gnomAD_v2.1, http://gnomad.broadinstitute.org, accessed on 3 May 2019) (Figure 1C). Two of the identified exonic variants in our cohort were missense SNPs (rs200149939, p.R185C; rs200746820, p.S213R). Overall, of the 34 detected genetic variants, one variant has not been described before in dbSNP. This novel variant, which is located in an intronic region, was found by NGS and was confirmed by Sanger re-sequencing (Supplementary Figure S2). A complex linkage pattern was found for several variants in the gene locus (Figure 1D) and apart from the reference haplotype we observed 7 further haplotypes with a frequency ≥1%, when including 17 common variants with MAF ≥ 5% in the haplotype block analysis (Supplementary Table S3). 

### 2.3. Impact of SLC10A1 Genetics on SLC10A1/NTCP Expression

We next performed a correlation analysis between the identified SNPs and *SLC10A1* mRNA expression as well as NTCP protein levels. Since 12 variants were only found heterozygously in one or two individuals (MAF < 1%), their influence on expression levels was not statistically tested. However, we observed increased or decreased expression levels compared to the median consistently across both expression phenotypes for the INDEL rs770421447 and the SNP rs186343960 (Supplementary Figure S3). The analysis for the variants with MAF > 1% (*n* = 22, Supplementary Figure S4) showed significantly decreased mRNA levels for rs111500198 (*P*_dominant_ = 0.04). The latter association was still significant in the multivariate analysis correcting for all seven non-genetic factors. Additionally, we observed two further significant associations for rs11622925 and rs4646285 with mRNA (*P*_recessive_ = 0.03) and rs76385306 with protein (*P*_additive_ = 0.02) expression in the multivariate analysis. However, all of these associations were no longer significant after adjustment for multiple testing. In addition, there were no further significant associations between genetic variants and NTCP protein levels in the univariate as well as a multivariate analysis correcting for all non-genetic factors. Moreover, no significant association was found between the estimated *SLC10A1* haplotypes and altered *SLC10A1* and NTCP expression (Supplementary Table S3).

### 2.4. Genetic Variability and Expression of SLC10A1 in Human Livers of the Cancer Genome Atlas 

The impact of genetic variants on *SLC10A1* gene expression was additionally independently evaluated in adjacent non-tumor tissues (*n* = 50) of the liver hepatocellular carcinoma (LIHC) cohort of The Cancer Genome Atlas (TCGA). The analyses confirmed the observation that genetic variants in the *SLC10A1* gene region are rare with no remarkable effects on gene expression levels (Supplementary Table S4).

### 2.5. Impact of SLC10A1 Missense Variants on SLC10A1/NTCP Function

**Figure 2:**
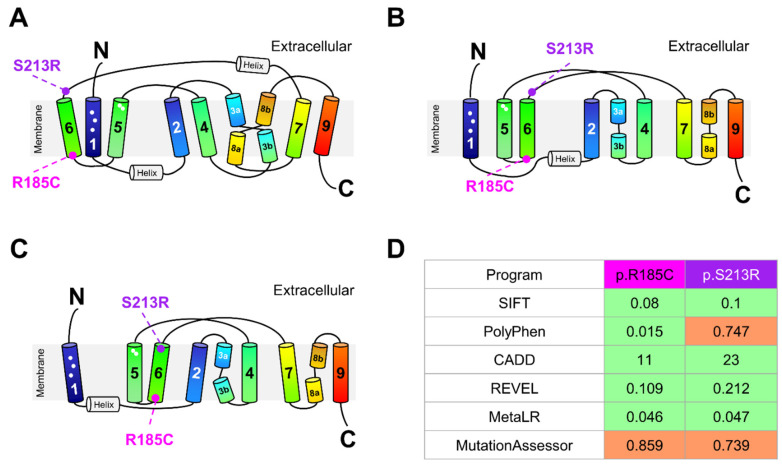
In silico analysis of NTCP missense variants. (**A**–**C**) Location of p.R185 and p.S213 in the three recently reported human NTCP structures. In all three structures, p.R185 is located at the start of the transmembrane segment (TM) 6. Amino acid S213 is located extracellularly in the topological models reported by (**A**) Goutam et al. [47] and (**B**) Asami et al. [46]. In the model reported by (**C**) Park et al. [48], this amino acid is located at the end of TM6. Putative conserved residues in the Na^+^-binding sites are located in TM2 (p.Q68), TM3b (p.S105/p.N106), TM4 (p.T123), and TM8a (p.E257, p.Q261); key residues for bile acid-binding are in TM1 (p.L27, p.L31, p.L35) and in TM5 (p.K157, p.G158) [48] and their approximate locations are indicated by white circles. TM1, TM5, and TM6 form the “panel domain” whereas the other TMs form the “core domain”. (**D**) Predicted functional consequences of p.R185C and p.S213R as obtained from Ensembl Release 106 (https://www.ensembl.org accessed on 6 June 2022). Green: tolerated; orange: possibly damaging.

In order to examine the functional influence of the only two missense variants (rs200149939, p.R185C; rs200746820, p.S213R), that were identified in our human liver cohort, we first determined the location of these variants in the recently reported NTCP models. Both variants are predicted to lie outside the bile acid-binding region (Figure 2A–C) and are classified as tolerated by algorithm SIFT, CADD, REVEL, and MetaLR while PolyPhen classified p.R185C as tolerated and p.S213R as possibly damaging and MutationAssessor classified both variants as possibly damaging (Figure 2D). Discrepancies in in silico predictions of NTCP variant function were also observed by Russell et al. [50] corroborating our findings that algorithms are limited to predicting NTCP function and in vitro functional assays are required.

**Figure 3:**
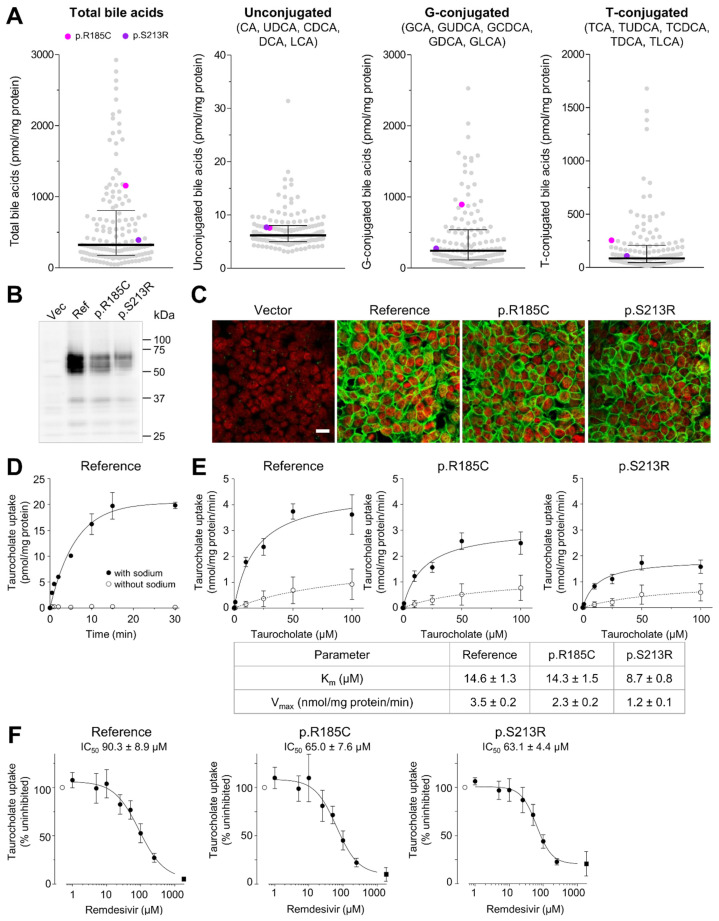
Functional characterization of NTCP missense variants in human liver and cell lines. (**A**) Box scatter plots show bile acid concentrations measured in liver cytosol using Q-TOF LC/MS. Individual levels are shown using gray colored circles. Heterozygous carriers of the two missense variants compared to non-carriers are illustrated by colored points: magenta, rs200149939, p.R185C; purple, rs200746820, p.S213R. The measured bile acids are CA: cholic acid, CDCA: chenodeoxycholic acid, DCA: deoxycholic acid, GCA: glycocholic acid, GCDCA: glycochenodeoxycholic acid, GDCA: glycodeoxycholic acid, GLCA: glycolithocholic acid, GUDCA: glycoursodeoxycholic acid, LCA: lithocholic acid, TCA: taurocholic acid, TCDCA: taurochenodeoxycholic acid, TDCA: taurodeoxycholic acid, TLCA: taurolithocholic acid TUDCA: tauroursodeoxycholic acid, UDCA: ursodeoxycholic acid. The lines indicate the medians. The whiskers correspond to the 25th and 75th percentiles. (**B**) Immunoblot analysis of protein lysates from HEK cells expressing NTCP reference sequence, p.R185C or p.S213R using affinity-purified NTCP-specific GNG antibody [58]. (**C**) Immunolocalization of NTCP (green) in vector-transfected control HEK cells and HEK cells stably expressing NTCP reference sequence or the respective missense variant using affinity-purified NTCP-specific GNG antibody [58] and confocal laser scanning microscopy. Red: nuclei. Bar, 20 µm. (**D**) Time-dependent uptake of 25 nM taurocholate (prototypic substrate) into HEK cells stably expressing NTCP reference in the presence (filled circle) or absence (open circle) of sodium. Results are means ± SD of 3 wells. (**E**) Concentration-dependent uptake of taurocholate into HEK cells stably expressing NTCP reference sequence or the respective missense variant in the presence (filled circle) or absence (open circle) of sodium determined at an incubation time of 30 s. Results are means ± SD of 12 wells. Kinetic parameters were calculated by subtracting taurocholate uptake in the absence of sodium from the uptake in the presence of sodium. (**F**) Inhibition of the uptake of prototypic substrate taurocholate by remdesivir into HEK cells expressing NTCP reference sequence or missense variant R185C or S213R. Cells were incubated with 15 µM taurocholate in the presence of different remdesivir concentrations and cellular accumulation was determined after 30 sec (filled circles). Results are presented as a percentage of uninhibited taurocholate uptake in the absence of remdesivir (100%, open circle). The filled square indicates taurocholate accumulation in the absence of sodium. Results are means ± SD from 9 wells.

We next measured bile acid concentrations of unconjugated, glycine-conjugated, and taurine-conjugated bile acids in the liver cytosol of the patients using mass spectrometry. As shown in Figure 3A, bile acid content does not appear to be altered in heterozygous carriers of the two missense variants compared to non-carriers. However, both variants were found only once heterozygously in the 143 liver samples limiting statistical analyses. We therefore stably expressed NTCP carrying either of the two missense variants in human embryonic kidney (HEK) cells. NTCP and the variant proteins were fully glycosylated (Figure 3B) and showed correct immunolocalization in the plasma membrane (Figure 3C). The prototypic substrate taurocholate was taken up by NTCP, p.R185C and p.S213R with comparable K_m_ values (Figure 3D,E).

Recently, the small molecule nucleoside analog remdesivir has been approved for the treatment of COVID-19 [59]. Because remdesivir was proposed as a novel inhibitor of NTCP [60] and transporter inhibition may be genotype-dependent [61], we analyzed whether both missense variants are also inhibited by remdesivir. The IC_50_ values were 90.3 µM, 65 µM, and 63.1 µM for NTCP reference, p.R185C and p.S213R, respectively (Figure 3F), and the R values calculated according to FDA guidelines [38] were 1.01 for NTCP reference and 1.02 for both variants. We then analyzed whether remdesivir is also a substrate of NTCP. We could identify a time- and sodium-dependent uptake of remdesivir into the NTCP transfectants (Supplementary Figure S5A). When analyzing the concentration-dependent uptake of remdesivir, we observed strong sodium-independent uptake into the transfectants (Supplementary Figure S5B). The sodium-dependent uptake was saturable with K_m_ values of 71.0 µM, 43.2 µM, and 219 µM for NTCP reference, p.R185C, and p.S213R, respectively. This data of high remdesivir uptake even in the absence of sodium indicates that NTCP or the two missense variants are not important determinants of hepatocellular remdesivir uptake.

### 2.6. Genome-Wide Association Study (GWAS)

**Figure 4:**
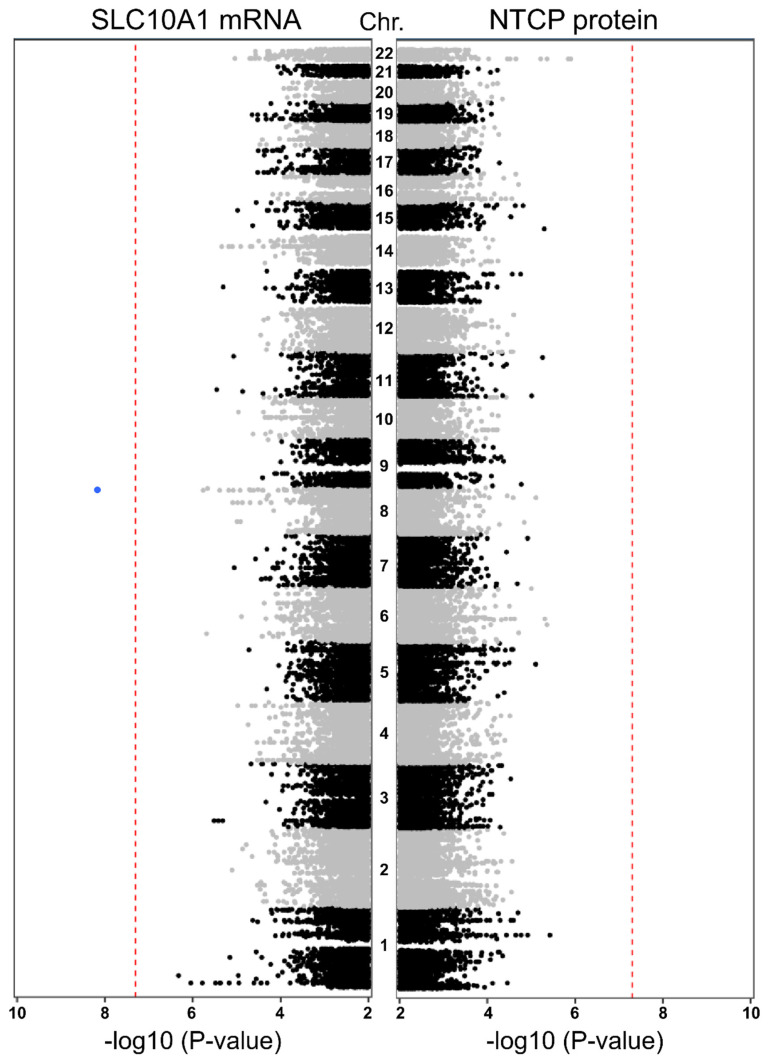
Genome-wide association analyses of genetic variants and *SLC10A1*/NTCP expression. Manhattan plots show the association of –log10-transformed *p*-values of genome-wide association analysis on *SLC10A1* mRNA (**left**) and NTCP protein level (**right**) across the 22 autosomes (from bottom [chr1] to top [chr22]) in the 143 liver samples. Red lines indicate the genome-wide significance level of *p*-value = 5 × 10^−8^.

To investigate other genomic regions altering *SLC10A1* expression we used imputed SNP data based on data generated using the human Infinium Global Screening Array v2.0 to investigate genome-wide associations between genetic variants and expression phenotypes. As shown in Figure 4, only one imputed variant on chromosome 8 was significantly associated with *SLC10A1* mRNA expression (chr8:133123929; rs2469637; MAF_this study_ = 0.44; MAF_EUR (Ensembl)_ = 0.41; *p* = 6.77 × 10^−9^; beta = −0.11). This variant is located in an intergenic region between the genes *HHLA1* and *KCNQ3* (potassium voltage-gated channel). However, the association was not confirmed on protein level (Figure 4) and no further associations of genetic variants neither with mRNA nor protein levels reached the genome-wide significance level of −log10[5 × 10^−8^]. 

### 2.7. Epigenetic Regulation of SLC10A1/NTCP

**Figure 5:**
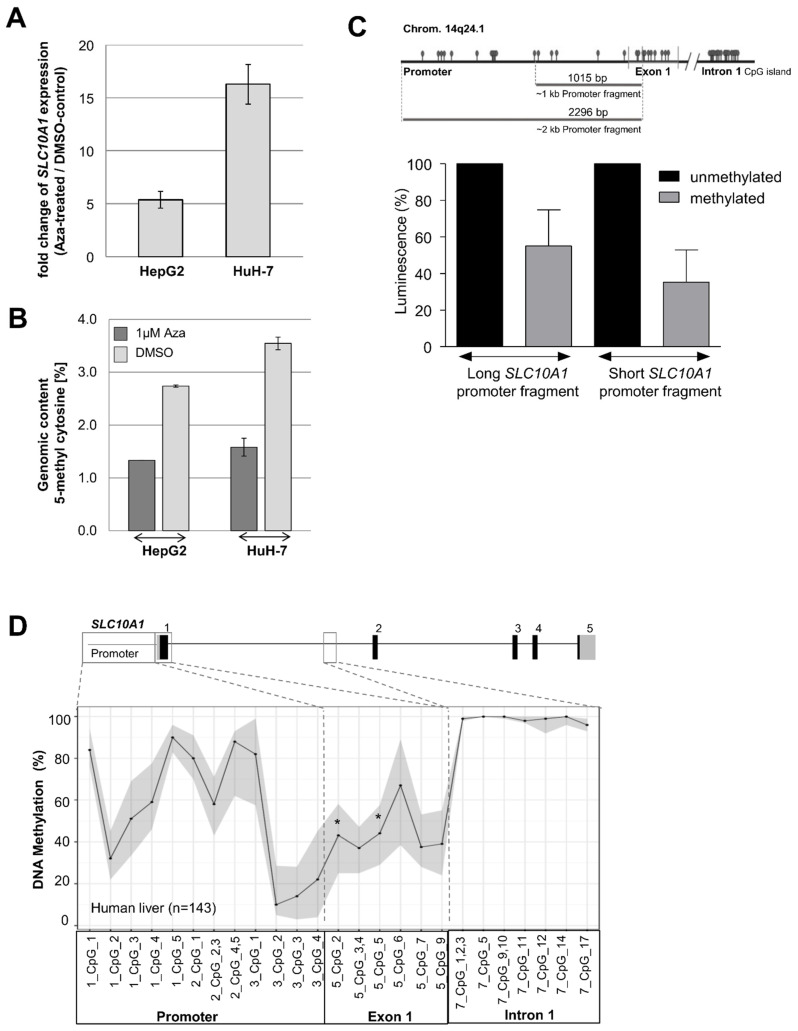
Epigenetic regulation of *SLC10A1*/NTCP. (**A**) Effect of 5-Aza-2’-deoxycytidine (AZA) treatment on *SLC10A1* mRNA expression. Cells were cultured with 1 µM AZA and mRNA levels (normalized to *β-actin*) were determined using TaqMan technology. Fold increase in expression compared to untreated cells was calculated. (**B**) Effect of treatment with 5-Aza-2’-deoxycytidine (AZA) on global DNA methylation. Cells were either untreated or treated with 1 μM AZA and the amount of 5-methylcytosine was quantified using LC-MS/MS to verify the effect of AZA treatment on global DNA methylation. Results represent mean of at least 2 experiments ± SE. (**C**) Scheme of the *SLC10A1* gene locus showing the examined promoter region. Two promoter fragments (short and long fragment), were cloned and reporter activity depending on methylation status of the promoter fragments was investigated in HuH-7 cells. Relative activities of the methylated fragments are shown compared to the mock-methylated fragments, whose activities were set to 100%. Experiments were performed in triplicates. (**D**) DNA methylation at individual CpG sites in the promoter, exon 1, and intron 1 gene region in human liver was quantified using MALDI-TOF MS. CpG sites significantly associated with protein levels even after correction for multiple testing are marked by an asterisk (*).

Because genetic variants seem to have only a minor impact on *SLC10A1*/NTCP expression and function, we next evaluated the impact of DNA methylation. First, functional evaluation of the epigenetic regulation of *SLC10A1*/NTCP expression was performed using cell culture experiments. HepG2 and HuH-7 cells were treated with AZA (5-Aza-2′-deoxycytidine, decitabine), which is a well-established DNA methylation inhibitor. Subsequently, mRNA expression of *SLC10A1* was analyzed using real-time PCR technology. As shown in Figure 5A, treatment of both HepG2 and HuH-7 cells with 1 µM AZA led to an increase of *SLC10A1* mRNA expression. Calculated as a multiple of the DMSO control, the AZA treatment led to a 5.4-fold elevation of the *SLC10A1* transcript level in HepG2 cells and a 16.3-fold increase in HuH-7 cells. In addition, global DNA methylation status was determined using an established liquid chromatography with tandem mass spectrometry (LC-MS/MS) method [62] in order to verify the demethylating effect of AZA treatment. As indicated in Figure 5B, the proportion of 5-methylcytosine residues in genomic DNA of HepG2 and HuH-7 cells decreased after treatment. 

To verify the influence of DNA methylation on *SLC10A1* promoter activity, different promoter/reporter fusion plasmids containing either methylated or mock-methylated *SLC10A1* promoter fragments were investigated (Figure 5C). Reporter gene constructs, in which the firefly luciferase gene was under the control of ~1 kb or ~2 kb *SLC10A1* promoter fragments, were transfected into HuH-7 cells. The obtained luciferase intensities of the lysates originating from cells transfected with unmethylated promoter-reporter gene constructs were set as 100%. As shown in Figure 5C, DNA methylation resulted in a reduction of luminescence of 44.8% for the methylated ~2 kb promoter fragment. For the methylated ~1 kb promoter fragment, the luminescence was reduced by 64.7%. Thus, reporter constructs with a methylated *SLC10A1* promoter fragment showed significantly reduced promoter activity compared to constructs with an unmethylated promoter, confirming the influence of DNA methylation on *SLC10A1* promoter activity.

Based on the results of the cell culture experiments, *SLC10A1* DNA methylation levels of individual CpG sites in the promoter and exon 1 region, as well as in a predicted CpG island of intron 1, were investigated using MALDI-TOF MS. As shown in Figure 5D, the DNA methylation was highly variable in the *SLC10A1* promoter and exon 1 gene region, whereas the CpG island in intron 1 was hypermethylated in human liver. Correlation analyses of DNA methylation levels and mRNA or protein levels (Table 2) indicated a significant association between DNA methylation at specific CpG sites and the protein levels even after Benjamini-Hochberg correction for multiple testing (5_CpG_5, adjusted *p* = 0.03; 5_CpG_2, adjusted *p* = 0.03).

### 2.8. Genome-Wide Expression Correlation Analysis and Gene Set Enrichment Analysis

**Figure 6:**
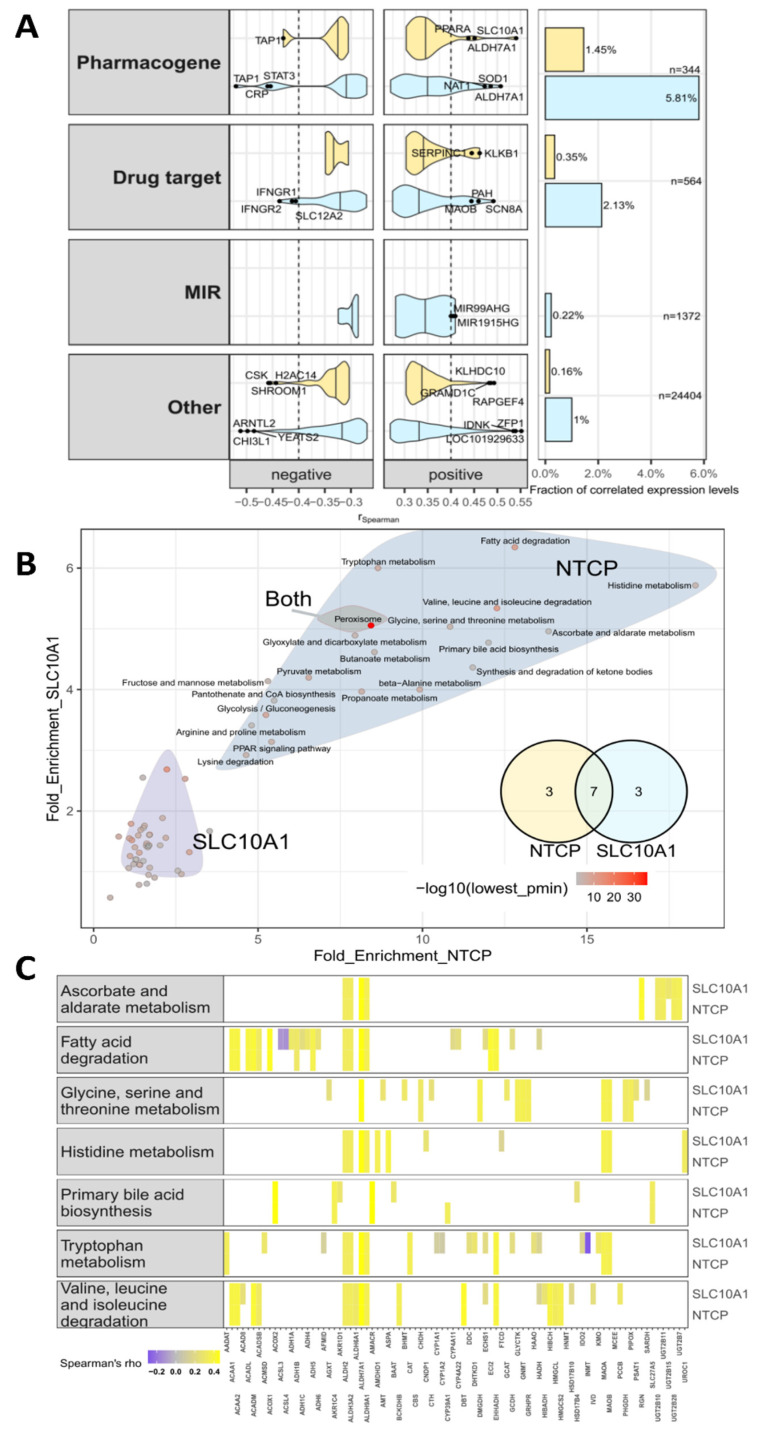
Genome-wide expression correlation analysis and gene set enrichment analysis. (**A**) Violin plots showing correlation results between genome-wide expression of the selected gene groups and *SLC10A1* expression levels as determined by qPCR (light blue) and NTCP protein levels (yellow), respectively. Top correlated genes (r_S_ ≤ −0.4 or r_S_ ≥ 0.4) are labeled with gene names. Barplots of the right figure are showing the percentage of significantly correlated genes (adjusted *p* < 0.05 and r_S_ ≤−0.4 or r_S_ ≥ 0.4). Values are stratified by selected gene groups in both plot types. (**B**) KEGG pathway enrichment analysis of correlation results of *SLC10A1* mRNA and NTCP protein levels using R package pathfindR. The gradient color reflects the lowest enrichment p value. Colored polygons indicate gene sets summarized using hierarchical clustering. The two top clusters are shown for *SLC10A1* and NTCP protein. The peroxisome pathway is among the top clusters for both, *SLC10A1* and NTCP protein. Overlap of the top 10 enriched terms for mRNA and protein are shown in the Venn diagram. (**C**) Heatmap visualizes the genes that are involved in the seven overlapping enriched pathways. The color gradient shows the Spearman correlation estimates.

In order to identify genes and pathways that are associated with NTCP expression, transcriptome and genes set enrichment analyses were performed using 118 liver tissues from our cohort with available genome-wide expression data. 1% and 0.1% of analyzed probesets comprising genes, pseudogenes and precursor microRNAs (*n* = 32,232) were significantly correlated (absolute r_S_ > 0.04) to *SLC10A1* mRNA expression (measured by qPCR) and NTCP protein levels, respectively. Then, we investigated whether pharmacogenes (*n* = 344) [63], target genes (*n* = 564) [64] or precursor miRNAs (*n* = 1372) were correlated with NTCP expression. The significantly correlated genes (adjusted *p* < 0.05, absolute r_S_ > 0.4) predominately belonged to the pharmacogene group (*SLC10A1* mRNA = 5.81%; NTCP protein = 1.45%; Figure 6A). Compared to the other groups, this overrepresentation was significant (*p* < 0.05). Both *SLC10A1* mRNA expression and NTCP protein levels were significantly positively correlated with ALDH7A1 expression (*SLC10A1*: r_S_ = 0.45; NTCP: r_S_ = 0.5), whereas highest negative correlation was found with ATP-binding cassette subfamily B member 2 (TAP1, r_S_ = −0.52). Interestingly, also transcription factors and nuclear receptors correlated to NTCP protein levels (e.g., *NR1I3* [CAR] r_S_ = 0.44), *PPARA* (r_S_ = 0.44), *STAT3* r_S_ = −0.45). Within the group of precursor miRNAs the highest significant correlation was found for MIR99AHG (r_S_ = 0.41). 

We next selected gene signatures from the Kyoto encyclopedia of genes and genomes (KEGG), gene ontology (GO) database, and metabolic signatures published in a study by Gaude et al. [65]. Gene set enrichment analyses were performed and revealed metabolic pathways associated with *SLC10A1* mRNA expression and NTCP protein levels. In Figure 6B the two top clusters for enriched pathways are shown for *SLC10A1* mRNA and NTCP protein. While the peroxisomal pathway was enriched for both mRNA and protein, the second clusters were different between both. Seven pathways (e.g., ascorbate, glycine, histidine, and tryptophan metabolism as well as fatty acid degradation) out of the top 10 enriched pathways (Figure 6B,C) were associated with *SLC10A1* mRNA expression and NTCP protein levels. Interestingly, mRNA expression and NTCP protein levels were significantly associated with the bile acid biosynthesis pathway. In addition, several other significant associations with metabolic pathways (e.g., valine, leucine, isoleucine metabolism) were found using also GO and metabolic signatures (Supplementary Figure S6).

### 2.9. SLC10A1/NTCP Expression and Hepatic Metabolism

**Figure 7:**
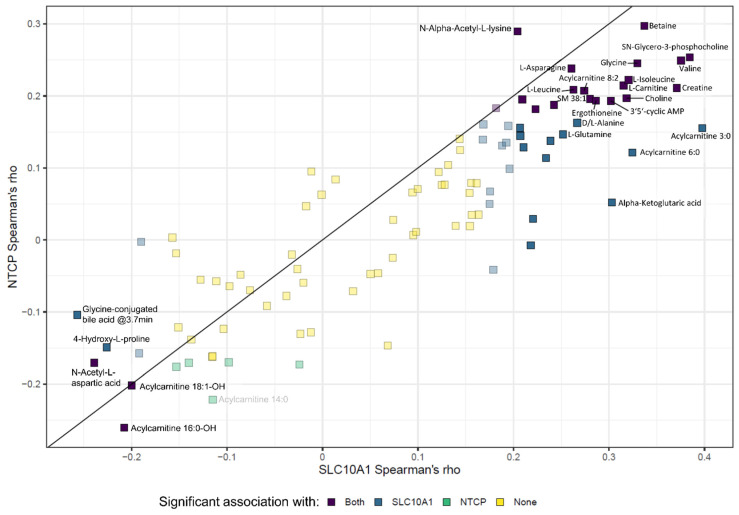
*SLC10A1*/NTCP expression and hepatic metabolism. Spearman correlations between mRNA expression and protein levels and endogenous metabolites in the 143 liver samples. *SLC10A1* mRNA expression was measured by qPCR and protein abundance was analyzed through LC-MS/MS. Metabolite levels were quantified using LC-MS technology. Correlations higher than 0.25 or lower than −0.2 are labeled, whereas correlations not significant after adjustment for multiple testing using the Benjamini-Hochberg procedure are only weakly colored.

Based on the results of our gene set enrichment analyses and the association with metabolic pathways, we further investigated whether the observed inter-individual variability of *SLC10A1*/NTCP expression was linked to altered endogenous metabolite levels in the liver tissues. Therefore, untargeted metabolomics analysis was performed as previously described [66]. Correlation analyses of metabolite levels and either mRNA expression or protein levels indicated significant associations with specific metabolites (Figure 7 and Supplementary Table S5) quantified in corresponding liver tissue. The highest correlation was observed for betaine even after correction for multiple testing. However, no NTCP-dependent transport of betaine was detected in HEK cells recombinantly expressing NTCP (Supplementary Figure S7). Moreover, certain acylcarnitines, amino acids as well as cyclic AMP (cAMP) were significantly correlated with both mRNA expression and protein levels of *SLC10A1*/NTCP (Figure 7).

In addition, the association of genetic variants within the *SLC10A1* gene region and metabolite levels was investigated. As shown in Supplementary Table S6, significant associations of certain variants were detected in univariate and multivariate analyses. However, only the associations with 4-hydroxy-L-proline showed a trend of significance (*p* = 0.05) after adjustment for multiple testing.

## 3. Discussion

Herein, we provide evidence that hepatic *SLC10A1*/NTCP expression is subject to a considerable inter-individual variability caused by various factors. In agreement with previous data which we had described for the hepatic uptake transporters *SLC22A1*/OCT1 [67] and *SLCO1B1*/OATP1B1 [56] cholestasis, alcohol consumption, and inflammation determined by C-reactive protein altered *SLC10A1*/NTCP expression whereas age and gender are of minor importance. Moreover, it cannot be excluded that protein expression/function may be altered by disease status as previously described [57,68,69]. Our comprehensive analyses of genetic variants in the promoter, the coding as well as 3′UTR/5′UTR of *SLC10A1* indicated that especially missense variants seem to be uncommon in Caucasians, which is consistent with a recent study [50] and was confirmed when considering population frequencies from gnomAD (Supplementary Figure S8). Cluster analysis based on genome-wide genetic data indicates that all individuals of our cohort are of European ancestry (Supplementary Figure S9). Using deep sequencing of all exons and intronic flanking regions, only two non-synonymous variants (rs200149939, p.R185C; rs200746820, p.S213R) were identified heterozygously in single individuals of our population. Population frequencies from gnomAD indicate that both missense variants are also only rarely detected in other ethnic populations, e.g., in the East Asian (rs200149939, MAF = 0.01%), African (rs200149939, MAF = 0.004%; rs200746820, MAF = 0.004%) or South American population (rs200149939, MAF = 0.003%; rs200746820, MAF = 0.006%) [70]. Since we did not observe a remarkable alteration in hepatic bile acid levels as endogenous NTCP substrate in individuals carrying these variants, a substantial functional effect for bile acid transport appears to be unlikely. However, the bile acid levels determined in our liver tissues might not only consist of intracellular bile acids but might additionally include canalicular or ductal bile acids [71]. We, therefore, generated HEK cells recombinantly expressing the variants p.R185C or p.S213R. Using these cells, we confirmed that both variants do not affect the bile acid transport function of NTCP.

In summary, none of the identified genetic variants in the *SLC10A1* gene region could explain the observed inter-individual variability in hepatic *SLC10A1*/NTCP expression. This could be confirmed using *SLC10A1* gene expression and germline genetic data from adjacent non-tumor liver tissue from the hepatocellular cohort of TCGA. In addition, our GWAS did not reveal any genetic variants in other genomic loci that are significantly associated with either mRNA expression or protein levels in our liver cohort to explain the inter-individual variability. 

Thus, epigenetic factors (e.g., DNA methylation), identified in our study might be more important for the regulation of hepatic *SLC10A1*/NTCP expression. We demonstrated that DNA methylation indeed had an impact on the regulation and expression of NTCP in vitro and in vivo. *SLC10A1* promoter activity was significantly influenced by DNA methylation and treatment of cells with the demethylating agent AZA led to an increase in *SLC10A1* mRNA expression. In the liver cohort, DNA methylation levels of certain CpG sites located in the proximal promoter region and in exon 1 in front of the translational start site were significantly associated also with NTCP protein levels and only to a lesser extent with *SLC10A1* mRNA. Of course, a direct effect of DNA methylation on transcriptional activity is expected, but the observed discrepancies between mRNA and protein (Table 2) could be due to different *SLC10A1* transcripts in liver tissue that were not quantified in our study. As shown in Supplementary Figure S1, a significant correlation between mRNA and protein was observed, which might be even higher considering all *SLC10A1* transcripts present in the liver. 

Moreover, other factors might influence mRNA expression and protein abundance. We found that the expression of the precursor miRNA MIR99AHG was significantly correlated with NTCP expression (Figure 6A). This precursor miRNA, which is also affiliated to long intergenic non-protein coding RNA, contains the miR-99a/let-7c/miR-125b-2 cluster. Interestingly, MIR99AHG was described recently as a tumor suppressor gene in lung adenocarcinoma [72] and was associated with advanced tumor progression and poorer prognosis in gastric cancer [73]. Moreover, MIR99AHG served as an oncogene in acute megakaryoblastic leukemia [74]. In silico analysis suggested only for let-7c a poorly conserved binding site in the *SLC10A1* 3’UTR using miRNAs target prediction tools (mirdb.org, targetscan.org, PolymiRTS). However, hepatic let-7c expression [75] did not correlate with NTCP expression, suggesting that the positive correlation of MIR99AHG with NTCP expression is due to other indirect effects, which need further investigation.

In addition, regulation by transcription factors might explain variability in mRNA expression. For instance, regulation by interleukin 1β via decreasing binding of HNF1α to its promoter was described for mouse *Slc10a1* [76] and NRF2 has been identified as a transcriptional regulator of *SLC10A1* [77]. However, neither *HNF1α* nor *NRF2* mRNA expression levels (determined in our transcriptome analyses) significantly correlated with either *SLC10A1* mRNA expression or NTCP protein levels. In contrast, we observed a significant correlation between the constitutive androstane receptor (CAR; *NR1I3*) and *PPARa* and *SLC10A1*/NTCP expression. The regulative role of PPARa in bile acid metabolism including hepatic NTCP expression changes upon PPARa activation has been shown in mice [78,79]. However, these results are contradictory as for instance different activators of PPARa increased or decreased NTCP expression levels. Our data underlines the contribution of PPARa but suggests also CAR as well as PXR (*NR1I2*) and LXRa (*NR1H3*; r_S_ > 0.35, *p* < 0.5) as direct or indirect regulators of NTCP expression in humans. 

The downregulation of NTCP associated with high CRP as a marker for inflammation mediated by cytokines is further supported by the negative correlation between STAT3 and *SLC10A1* mRNA levels in our study since STAT3 regulates several genes involved in the inflammatory response [80]. In line with this, recently a STAT3-mediated downregulation of hepatic transporters including *SLC10A1* has been shown in mice [81].

Generally, it can be assumed that NTCP has an influence on cellular hepatic metabolism because of its role in the uptake of important endogenous substances, like bile acids. However, NTCP also undergoes a complex post-translational regulation process mediated by metabolic signaling pathways which seem to be species-specific [1]. We, therefore, performed a gene set enrichment analysis using transcriptome data, which indicates that NTCP protein abundance is significantly associated with peroxisomal and fatty acid degradation, as well as bile acid biosynthesis pathways. The association with the peroxisomal pathway is supported by a study by Keane et al. [82]. Here, NTCP protein levels were impaired in PEX2 mutant mouse livers. Generally, NTCP expression levels seem to be majorly associated with metabolic pathways in the liver. This observation is supported by a recent study by Zhang et al. [83], who showed that several pathways, such as that of tyrosine, glycine, taurine, fatty acids, and glycerophospholipids metabolism, are dysregulated in NTCP knockout mice. 

Therefore, we investigated the relationship between *SLC10A1*/NTCP expression and endogenous metabolism using an untargeted metabolomics approach. A significant correlation of both mRNA expression and protein levels with certain metabolites like betaine or specific acylcarnitines was observed. The highest correlation, which was significant even after correction for multiple testing, was found for betaine, an osmolyte with an important role as methyl donor in hepatic metabolism [84]. Because betaine is not an NTCP substrate (Supplementary Figure S7), we postulate that the observed correlation is indirect and results from the fact that NTCP expression correlates with that of ALDH7A1, which metabolizes aldehyde substrates, including betaine aldehyde to betaine [85]. The underlying mechanism of the relationship between NTCP and ALDH7A1 needs further investigation. Additionally, we identified a correlation with cAMP levels. cAMP has previously been shown to increase plasma membrane levels of NTCP in rat hepatocytes and in hepatic cells transfected with human NTCP [86,87].

In conclusion, we could demonstrate that genetic variation of *SLC10A1* cannot explain the inter-individual variability of NTCP and its function. In contrast, non-genetic (i.e., smoking, alcohol consumption) and epigenetic factors, as well as endogenous metabolites, contribute to the inter-individual variability of NTCP indicating the complexity of expression regulation of human transporter proteins.

## 4. Materials and Methods

### 4.1. Liver Tissue Samples

A total of 143 well-characterized, non-tumor and HBV-free human liver samples, as well as corresponding blood samples, were collected from patients undergoing liver surgery due to liver metastasis, hepatocellular carcinoma, cholangiocellular carcinoma, haemangioma, Caroli syndrome, gallbladder carcinoma, liver cysts at the Department of General, Visceral and Transplantation Surgery (University Medical Center Charité, Berlin, Germany) as previously described [67]. The study cohort consists of 68 male and 75 female individuals with a median age of 58 years. Demographic and clinical characteristics are shown together with data on the association of non-genetic factors on *SLC10A1* mRNA expression and NTCP protein abundance in Supplementary Table S1. 29 of the patients were smokers (≥1 pack/day) and regular alcohol consumption (at least ≥1 times/week) was reported for 46 individuals. The median CRP level was 0.5 mg/L (range 0.1–21.1 mg/L). Cholestasis (yes vs. no) was defined as previously described [67]. The study was approved by the ethics committee of the University of Tuebingen (Tuebingen, Germany) in accordance with the principles of the Declaration of Helsinki.

### 4.2. Analysis of SLC10A1 Expression and Genetic Variability in the TCGA LIHC Cohort

For validation of data, we used the LIHC cohort of TCGA, for which whole-exome sequencing data of germline DNA and RNA-seq data of tumor-adjacent histologically non-tumor liver tissue (*n* = 50) is available. Analysis-ready bam files storing aligned (genome build hg38) whole-exome sequencing data from tumor-adjacent tissue as well as blood samples from TCGA LIHC cohort were downloaded from Genomic Data Commons Data Portal (https://portal.gdc.cancer.gov/, downloaded on 2 February 2019). Germline variants were called using the GATK software (version 4.1). Variant calling using HaplotypeCaller and variant quality score recalibration were performed as suggested in GATK Best Practices. Variant calling was confined to exome regions as defined in Agilent SureSelect Version 7 interval file (hg38). Gene expression data (“FPKM-UQ”) based on RNA-seq from TCGA LIHC cohort were downloaded from Genomic Data Commons Data Portal (https://portal.gdc.cancer.gov/, downloaded on 2 February 2019). FPKM-UQ values were log2-transformed before analysis. For 50 cases both expression data from tumor-adjacent tissue as well as germline variants were available. Association between *SLC10A1* gene expression and *SLC10A1* genotypes was analyzed by frequentist association tests as implemented in the SNPTEST software (version 2.5.2).

### 4.3. RNA Isolation and Quantification

High-quality RNA of liver samples was extracted as described previously [67]. For reverse transcription of the RNA the High-Capacity cDNA Reverse Transcription Kit with RNase Inhibitor (Applied Biosystems, Foster City, CA, USA) was used. A *SLC10A1* clone was created containing the complete cDNA in the pGEM T-Easy plasmid (Promega, Madison, WI, USA). The clone was verified by sequencing and used for absolute quantification of *SLC10A1* expression in the liver.

The *SLC10A1* cDNA amount was measured with the TaqMan^®^ Gene Expression Assay Hs_00161820_m1 (Applied Biosystems). The *SLC10A1* results were normalized against *β-actin* expression, which was measured with the HUMAN ACTB (beta actin) Endogenous Control Assay (Applied Biosystems). The measurements were conducted on the Fast Real Time PCR System 7900HT (Applied Biosystems).

### 4.4. LC–MS/MS-Based Targeted Proteomics

All material and chemicals for targeted proteomics were purchased from ThermoFisher Scientific (Waltham, MA, USA) or Sigma-Aldrich (St. Louis, MO, USA) unless otherwise stated.

#### 4.4.1. Sample Preparation and Digestion

Membrane protein-enriched fractions from human liver tissue were prepared as described previously [67]. Sample preparation and LC-MS/MS analysis were performed as described in Wegler et al., with minor modifications [88]. In brief, 200 µL of the membrane fractions were treated with DTT to a final concentration of 100 mM and boiled for 5 min. 20 µL 20% SDS was added and the samples were sonicated to ensure complete solubilization. Lysates were clarified by a 15 min centrifugation at 10,000× *g* and the clarified lysates were transferred to a new tube for further processing. The protein concentrations were determined using the tryptophan fluorescence assay [89]. Aliquots of the lysates containing 100 μg total protein were processed according to the filter-aided sample preparation protocol using the Microcon-30kDa centrifugal filter unit (Merck Millipore, Burlington, MA, USA) [90]. The lysates were depleted from detergents by successive washes with 8 M urea in 0.1 M Tris/HCl at pH 8.5. Thiols were alkylated with iodoacetamide (50 mM in 8 M urea in 0.1 M Tris/HCl at pH 8.5), and proteins were digested with sequencing grade modified trypsin (Promega), with an enzyme/protein ratio of 1:40, for 16 h. Digested peptides were eluted using 2.5% formic acid and quantified by tryptophan fluorescence. The digestion yields were determined and were always above 60%. Prior to LC-MS/MS analysis, the digests were evaporated to dryness and dissolved in 50% acetonitrile containing 0.1% formic acid, to a final concentration of 0.5 mg/mL.

#### 4.4.2. LC-MS/MS and Data Analysis

Peptide surrogates of analytical grade (>95% purity) were obtained from JPT (Berlin, Germany) and TAGC (Copenhagen, Denmark). The peptide digests were spiked with stable-isotope labeled (SIL) peptides (Supplementary Table S7). Four μg of peptides from the peptide digests containing the SIL peptide were separated on a C18 reverse-phase column (Acquity UPLC BEH C18 1.7 µm, 2.1 × 100 mm) using a 19 min gradient of water and acetonitrile with 0.1% (*v*/*v*) formic acid in an Agilent 1290 Infinity binary LC system. Three mass transitions per peptide were quantified using the scheduled multiple reaction monitoring mode in a QTRAP 6500 mass spectrometer (AB Sciex, Framingham, MA, USA) in electrospray positive mode. Mass transitions are listed in Supplementary Table S7. The calibration curve was linear throughout the calibration range with a correlation coefficient (r) higher than 0.99. Quality controls were run between every eight samples and a maximal divergence of less than 15% from the actual concentration was observed. The lower limit of quantification was 0.1 fmol/μg total protein. Targeted proteomic data was analyzed using Analyst 1.6.2 and MultiQuant 3.0.

### 4.5. SLC10A1 Sequencing and Genotyping

Purification of genomic DNA from EDTA blood samples was performed as described previously [67]. The *SLC10A1* gene region (chr14:70,242,552..70,264,006; hg19) was analyzed systematically in the 143 individuals for the presence of genetic variations by NGS, Sanger sequencing, MALDI-TOF MS, and microarray technology. The analyzed region covered 2 kb promoter region, all exonic, as well as flanking intronic regions, and the 5′UTR and 3′UTR regions. 

#### 4.5.1. Next Generation Sequencing (NGS)

NGS was conducted at the Center for Genomics and Transcriptomics (CeGaT GmbH, Tuebingen, Germany) [63]. In brief, a custom-made ADME panel including *SLC10A1* was designed and manufactured for use with Agilent in-solution target capture technology (Agilent Technologies, Santa Clara, CA, USA). Target regions of *SLC10A1* used for library preparation are given in Supplementary Table S8. The DNA libraries were generated from the genomic DNA of 143 blood samples. NGS was performed at a high depth (459-fold mean coverage) with 2 × 100 bp paired-end reads on the Illumina HiSeq2500 platform (Illumina Inc., San Diego, CA, USA). Raw reads were mapped to the human reference genome (hg19) and variations were called and annotated using a custom bioinformatics pipeline, also covering previously published biomarkers. Filtering of germline variants was performed as described previously [91].

#### 4.5.2. MALDI-TOF MS-Based Genotyping

28 known *SLC10A1* variants were genotyped using MALDI-TOF MS. These comprised all variants described in the literature, as well as promoter and missense exonic variants with minor allele frequencies (MAF) ≥ 0.5% or unknown MAF in Caucasians. Additionally, two missense variants were included, which were located in the first described region important for HBV binding (NTCP p.157–164) [41], rs201339654 and rs199663299. For assay design, the MassARRAY^®^ Assay Design v.3.1 program (Sequenom/Agena Bioscience, San Diego, CA, USA) was used.

Regions of interest were preamplified in 4 multiplex-PCRs using 10 ng of DNA as a template. The iPLEX^®^ Gold Reagent Kit (Sequenom/Agena Bioscience) was used for the following allele-specific primer extension reaction. Samples were measured on the MassARRAY^®^ Compact System MALDI-TOF MS (Sequenom/Agena Bioscience). For quality control, 10% of the samples were run as duplicates. Information about primer sequences is available upon request.

#### 4.5.3. Sanger Sequencing

As an additional control, 12 samples with particularly high or low *SLC10A1* expression were sequenced by Sanger sequencing in the above-mentioned regions. Variants newly identified by NGS were also confirmed by Sanger re-sequencing of the respective samples. The regions of interest were preamplified from 20 ng of genomic DNA. For the following sequencing-PCR the BigDye Terminator v1.1 Cycle Sequencing Kit (Applied Biosystems) was used together with 30–50 ng of the preamplification product and 3.3 pmol of primers. The products were separated and detected by capillary gel electrophoresis on the ABI3500 DX System (Applied Biosystems). Primer sequences and conditions are provided upon request.

#### 4.5.4. Genome-Wide SNP Microarray

Additionally, information on 5 *SLC10A1* SNPs of the HumanHap300 Genotyping BeadChip (Illumina Inc., San Diego, CA, USA) was available [92]. Moreover, whole-genome genotyping of more than 700k SNPs was performed using the Infinium™ Global Screening Array-v2.0 (Illumina Inc.).

### 4.6. Location of NTCP Variants in the Human NTCP Structure and In Silico Prediction of Their Functional Effects

The variants p.R185C and p.S213R were located on the three recently reported NTCP structures [46,47,48]. Functional effects of both variants were predicted using the following 6 different algorithms as provided by the Ensembl Genome Browser (https://www.ensembl.org, release 106, last accessed on 6 June 2022): Sorting Intolerant From Tolerant (SIFT), Poly-Phenotyping-2 (PolyPhen-2), Combined Annotation-Dependent Depletion (CADD), Rare Exome Variant Ensemble Learner (REVEL), Meta Logistic Regression (MetaLR) and MutationAssessor. Variants were considered as tolerated when scores were ≥0.05 (SIFT), ≤0.5 (PolyPhen, REVEL, MetaLR, MutationAssessor) or <30 (CADD). 

### 4.7. Generation and Characterization of Cell Lines Overexpressing NTCP and NTCP Variants

DNAs either encoding the NTCP reference sequence (NM_003049), NTCP-p.R185C (rs200149939) or NTCP-p.S213R (rs200746820) were synthesized and each cloned into the pcDNA5/FRT FlpIn vector (ThermoFisher Scientific) by BioCat GmbH (Heidelberg, Germany). These expression vectors were used to generate stably transfected FlpIn HEK cells (ThermoFisher Scientific) as described [91]. HEK cells were cultivated in DMEM supplemented with 10% FBS, 100 U/mL of penicillin, and 100 μg/mL of streptomycin (Lonza, Basel, Switzerland) at 37 °C and 5% CO_2_.

NTCP proteins in transfectants were detected by GNG antibody. Generation of the polyclonal GNG antiserum against NTCP has been described [58]. The GNG antiserum was affinity-purified by ImmunoGlobe GmbH (Himmelstadt, Germany) using the NTCP-specific peptide used for generation of the antiserum. Immunoblot analysis was carried out as described [91] using 20 µg of protein lysate of the transfectants and the affinity-purified GNG antibody at a 1:1000 dilution. Immunofluorescence staining of NTCP transfectants was carried out as described [93] with affinity-purified GNG antibody diluted 1:100 in PBS. 

### 4.8. Uptake Studies

Chemicals for uptake studies were from Merck KGaA (Darmstadt, Germany) if not stated otherwise. Uptake studies with the prototypical substrate taurocholate, with remdesivir or betaine were carried out at 37 °C as described [93] either in the presence of sodium or in the absence of sodium with choline chloride as replacement of NaCl. Taurocholate uptake was measured in the presence of a tracer amount of 25 nM [^3^H(G)]-taurocholic acid (740 GBq/mmol; American Radiolabeled Chemicals Inc., St. Louis, MO, USA) and different concentrations of unlabeled taurocholate. Remdesivir (Sellekchem, Houston, TX, USA) uptake was determined by UHPLC-MS/MS as described [94]. Time-dependent uptake of taurocholate and remdesivir was assessed at a concentration of 25 nM and 5 µM, respectively. Kinetic parameters K_m_ and V_max_ were determined within the linear uptake ranges, i.e., 30 s for taurocholate and 10 min for remdesivir. For the determination of IC_50_ values of remdesivir for inhibition of taurocholate uptake, 7 different remdesivir concentrations between 1 µM and 250 µM were used. Betaine accumulation was assessed at a concentration of 100 nM betaine [glycine-2-^3^H] (740 GBq/mmol, American Radiolabeled Chemicals Inc.) at 1 and 10 min.

Transport data were analyzed with GraphPad Prism 9.3.0 (GraphPad Software Inc., La Jolla, CA, USA). Time-dependent uptake values of taurocholate and remdesivir were fitted by non-linear regression (least-squares fit) to the following equation as described: [91] c_in_ = k_in_/k_out_ × c_out_ × [1 − exp(−k_out_ × t)] with k_in_ and k_out_ being the rate constants for inwardly- and outwardly-directed transport, respectively, c_in_ and c_out_ being the intracellular and extracellular substrate concentrations, respectively, and t being the time. For determination of the kinetic parameters K_m_ and V_max_, uptake values were at first corrected for uptake in the absence of sodium. Data were then fitted by weighted (1/y^2^) non-linear regression, using the Michaelis-Menten equation: V = (V_max_ × [S])/(K_m_ + [S]) with V being the initial uptake velocity and [S] the substrate concentration. The remdesivir concentrations that achieved half-maximum inhibition (IC_50_) of taurocholate accumulation were also determined by non-linear regression fitting the values to the following 4-parameter equation: uptake(S) = uptake(min) + (uptake(min) − uptake(max))/(1 + (([S]/IC_50_)^slope^)) with uptake(S) being the taurocholate uptake at a given remdesivir concentration [S], uptake(min) and uptake(max) the minimal and maximal taurocholate uptake values, respectively, and slope the Hill slope. Taurocholate uptake in the absence of sodium was considered as the minimal uptake and used as a constraint in the calculation of the IC_50_ values.

### 4.9. DNA Methylation Analyses Using MALDI-TOF Mass Spectrometry

For quantitative DNA methylation analyses, MALDI-TOF MS was applied as previously described [57,95]. In brief, after bisulfite treatment of the genomic DNA, amplicons covering the *SLC10A1* promoter and exon 1 region, as well as a CpG island in the intron 1 region were generated using primers for methylation-specific PCR amplification, which were designed with the software Methprimer (http://www.urogene.org/methprimer/index1.html). Primer sequences are provided upon request. Forward primers included a 10-mer tag and reverse primers were tagged with a T7 polymerase promoter allowing subsequent reverse transcription using the MassCLEAVE^TM^ reagent Kit (Sequenom/Agena Bioscience). Single-stranded RNA strands generated by reverse transcription were cleaved base-specifically by RNase A, resulting in fragments of defined length. Mass spectra were obtained using the MassARRAY compact system and evaluation of spectra methylation ratios was performed by use of the EpiTYPER 1.0 software.

### 4.10. Cell Culture Experiments and Treatment with 5-Aza-2′-deoxycytidine

For the in vitro methylation experiments human hepatocellular carcinoma cell lines HuH-7 and HepG2 were used. HepG2 cells were purchased from the European Collection of Cell Cultures (ECACC). Cell lines were routinely tested for mycoplasma infection using a PCR-based test (Venor^®^GeM Classic, Minerva Biolabs GmbH, Berlin, Germany). Cell line authentication was performed using the Power Plex^®^ 21 system (Promega) on the 3500DX System (Applied Biosystems) according to the manufacturer’s instructions.

HuH-7 and HepG2 cells were plated with a density of 2.5–3.4 × 10^4^ cells/cm^2^ and pre-incubated for 24 h prior treatment with 5-Aza-2′-deoxycytidine (AZA). For DNA demethylation experiments, the cells were treated with 1 µM AZA (Sigma-Aldrich) dissolved in DMSO or DMSO (Sigma-Aldrich) and incubated for 72 h. 5-methyl-2’-deoxycytidine (5-meC) content was determined using LC-MS/MS analysis as previously described. DNA methylation levels are given as percentage of 5-methyl-2’-deoxycytidine content relative to total cytosine residues [62].

### 4.11. SLC10A1 Promoter-Reporter-Gene Constructs

Two promoter fragments were constructed: A 2296 bp construct, containing the whole analyzed promoter region and the beginning of exon 1 up to the translational start site (−2173 up to +123). The other construct of 1015 bp contained the end of the promoter and the beginning of exon 1 (−892 up to +123). The primers used contained tags with recognition sequences for restriction enzymes: forward with KpnI, reverse with BglII. (SLC10A1 Prom_long_f: gctg-ggtacc-GGTGTCAGGTTCCTGGAAGA, SLC10A1 Prom_short_f: gctg-ggtacc- ATGCCCACCTCTCTCTGCT, SLC10A1 Prom_r: gcccagatct-GCAGTGGAAGACCACTCCTT).

One part of the promoter fragments was methylated using CpG methyltransferase M.SssI (New England BioLabs, Ipswich, MA, USA) in the presence of S-adenosylmethionine (SAM), the other part was mock-methylated under equal conditions in absence of CpG methyltransferase M.SssI. The promoter fragments were ligated into the pGL4.10 vector (Promega). HuH-7 cells were used for transfection with the constructs and the pRL-TK control vector (Promega) in a ratio of 30:1. Luciferase activity was measured using the Dual-Luciferase^®^ Reporter Assay System (Promega) according to manufacturer’s instructions. 

### 4.12. Quantification of Bile Acids 

Bile acids were quantified by LC-MS as described previously [96]. Briefly, 10 µL liver cytosol [97] (80–360 µg protein) was diluted with 40 µL water and subjected to methanol precipitation in the presence of deuterium-labeled internal standards (10 pmol per 50 µL sample volume). Calibration samples for the generation of calibration curves were processed in parallel with the following concentration ranges: 0.4–50 pmol for CA, CDCA, UDCA, DCA, LCA, TUDCA, GLCA, and TLCA and 0.8–100 pmol for GCA, TCA, GCDCA, TCDCA, GDCA, TDCA and GUDCA. 200 µL supernatant of each methanol-precipitated sample was transferred to a new tube, dried by a gentle stream of nitrogen, and re-suspended in 110 µL methanol:water (1:1, *v*/*v*) following LC-MS analysis. The precision and accuracy of the method were evaluated by analyzing quality control (QC) samples, prepared like the calibration samples. Bile acid quantitative data was normalized to protein amount. Concentration values, including values below the limit of quantification, were used for further statistical analysis [98]. 

### 4.13. Untargeted Metabolomics Analyses

Metabolomics analysis by ultra-high performance liquid chromatography-quadrupole time-of-flight mass spectrometry (UHPLC-Q-TOF-MS) was performed as previously described [66]. In brief, frozen liver tissue samples of approximately 12–60 mg were randomized to 8 batches for tissue homogenization and subsequent two-step metabolite extraction. QC samples were prepared by pooling equal volumes of extracts derived from 30 liver samples (QC pool) [99]. Dried aqueous metabolite extracts were reconstituted in water/acetonitrile (5:95, *v*/*v*) at a solvent/tissue ratio of 100 µL/mg and analyzed after HILIC separation in positive and negative ionization mode on a 1290 Infinity UHPLC System coupled to a 6550 iFunnel QTOF-MS (Agilent Technologies) equipped with a Dual Agilent Jet Stream electrospray source. Samples were measured in 6 analytical batches (3 positive and 3 negative ion mode) with a QC sample analyzed after every 5th patient sample. Data was preprocessed by targeted extraction of annotated features [66]. Peak areas were normalized by median-normalization for each sample following LOESS correction based on QC samples for individual features (intra-batch correction). For inter-batch correction, the median of QC samples was calculated for each feature and batch. The values of the samples in each batch were subsequently divided by the corresponding value. Features with CVs >20% within individual batches and features with significant fold-changes between batches (max. fold change > 1.5 and Bonferroni-adjusted Kruskal-Wallis test *p* > 0.05) were excluded. Normalization and filtering were performed separately for both the positive and negative ion mode. Finally, structural assignment of features was performed as previously described [66] based on the accurate mass (±15 ppm) and/or fragmentation patterns derived from MS/MS spectra. In summary, 98 metabolites were annotated and included in the statistical analyses.

### 4.14. Transcriptome and Gene Set Enrichment Analysis

For isolation of total RNA from fresh-frozen tissue, the mirVana™ miRNA Isolation Kit (Life Technologies) was used according to the manufacturer’s protocol. Large-scale gene expression profiling was performed using Human Transcriptome Array 2.0 (HTA2.0) (ThermoFisher Scientific) as previously described [100,101]. NTCP expression phenotypes were correlated to HTA2.0 data including 33,494 probe sets using Spearman’s rank correlation method. Gene symbols were annotated using the HTA2.0 Probeset Annotations Release 36 as well as biomart (https://www.ensembl.org/biomart, accessed on 23 September 2021, Ensembl Release 104).

*p*-values were adjusted for multiple testing using the Benjamini-Hochberg procedure. The adjusted correlation *p*-values, as well as the correlation estimates, were further analyzed using GO and KEGG databases as well as custom gene sets for metabolic pathways established by Gaude et al. [65] using R package pathfinder [102]. 

### 4.15. Statistics

Statistical analyses were performed using R-4.1.3 (http://www.r-project.org) [103] with the additional packages beeswarm_0.6.0 [104], tidyverse_1.3.1 [105], qqman_0.1.4 [106] and SNPassoc_2.0–11 [107]. Associations between demographic and clinical factors and *SLC10A1* mRNA expression and protein levels were investigated using Wilcoxon-Mann-Whitney tests or Spearman’s rank correlation coefficients as appropriate.

Univariate and multivariate association analyses between the genotyped variants and *SLC10A1* mRNA expression or protein levels were performed using the generalized linear model capabilities of the SNPassoc package. For this purpose, *SLC10A1* mRNA and NTCP protein measurements were first power-transformed (*SLC10A1*: λ = 0.33, NTCP: λ = 0.7) to fulfill Gaussian distribution assumption. The transformation was determined via the R-package car_3.0-2 [108]. Distributions of transformed measurements were confirmed using a quantile-quantile plot and Shapiro-Wilk tests. In multivariate analysis, we used seven demographic and clinical factors (gender, age, CRP, cholestasis, medication, alcohol, and nicotine consumption) as covariates. In addition, associations between the *SLC10A1* genetic variants and the bile acid concentrations were analyzed using Kruskal-Wallis tests.

Association study of genome-wide imputed genotypes was performed with SNPTEST v2.5 using the frequentist association test option with expected genotype counts. Imputation of the GSA-SNP array data was performed using Minimac3 software on the Michigan Imputation Server [109]. File preparation was performed using PLINK software and HRC preparation checking tool version 4.3.0 (https://www.well.ox.ac.uk/~wrayner/tools/). Vcf-files of autosomal SNPs were subject to imputation. HRC-Tool, eagle_v2.3 for phasing and Minimac3 were run using default parameters and hrc.r1.1.2016 haplotypes as reference panel. The association between each of the genetic variants and the transformed measurements was analyzed in the additive genetic model.

For the analyses of linkage disequilibrium and haplotypes the Haploview version 4.2 software (http://www.broadinstitute.org/haploview, Cambridge, MA, USA) was used. Hardy-Weinberg equilibrium calculations were performed to compare observed and expected allele and genotype frequencies. Regulatory motifs affected by genetic variants were identified using HaploReg version 4.1 (http://www.broadinstitute.org/mammals/haploreg/haploreg.php). 

Correlation coefficients between mRNA and protein levels and endogenous metabolites were determined using Spearman’s rho (r_S_). Associations between SNPs (MAF > 1%) and metabolites were investigated using Kruskal-Wallis test for univariate analysis as well as ANOVA for median regression for multivariate analysis. To be more precise, for each metabolite phenotype and each chosen SNP, we applied function anova.rq in R-package quantreg-4.91 with rank test-statistic and Wilcoxon score function to compare two median regression fits with only the seven non-genetic demographic and clinical factors as covariates versus a model of the seven non-genetic factors plus the considered SNP. All statistical tests were two-sided and statistical significance was defined as *p* < 0.05. Where indicated, adjustment for multiple testing was carried out using the Benjamini-Hochberg procedure [110]. 

## Figures and Tables

**Table 1:** Clinical parameters and extrapolations for NTCP inhibitor drugs.

Drug ^1^	Dose (mg)	I_max_ (µM) ^2^	I_in,max_ (µM) ^2^	f_U_ ^2^	I_in,max,u_ (µM) ^2^	IC_50_ (µM) ^3^	R ^4^
Bendroflumethiazide ^5^	10 [12]	0.2 [12]	1.68	0.06 [13]	0.101	77	1.00
Bosentan	125 [14]	4.2 [14]	18.2	0.02 [15]	0.365	24 [16]	1.02
Budesonide ^5^	0.8 [17]	0.0026 [17]	0.12	0.13 [13]	0.015	320	1.00
Bulevirtide (Myrcludex B)	2 [8]	0.04 [8]	0.07	0.85 [18]	0.056	0.053 [19]	2.06
Candesartan ^5^	32 [20]	0.75 [20]	5.26	0.01 [13]	0.053	339	1.00
Cyclosporin A	700 [21]	0.45 [21]	36.6	0.12 [21]	4.39	1 [22]	5.39
Cyclosporin A	200 [23]	1.8 [23]	12.2	0.1 [23]	1.22	1 [22]	2.22
Diltiazem ^5^	180 [21]	0.29 [21]	27.3	0.3 [21]	8.17	871	1.01
Diltiazem ^5^	120 [23]	0.36 [23]	18.3	0.22 [23]	4.04	871	1.00
Doxazosin ^5^	8 [24]	0.06 [24]	1.16	0.02 [24]	0.023	51	1.00
Efavirenz	600 [25]	11.6 [25]	129.7	0.01 [13]	1.3	43 [26]	1.03
Ezetimibe ^5^	10 [24]	0.01 [24]	1.53	0.1 [24]	0.153	36	1.00
Fenofibrate ^5^	300 [23]	23.8 [23]	75.5	0.01 [13]	0.755	188	1.00
Flutamide ^5^	250 [24]	0.4 [24]	56.6	0.06 [24]	3.4	164	1.02
Furosemide	80 [27]	6.7 [27]	21.7	0.01 [13]	0.217	15 [22]	1.01
Gemfibrozil ^5^	600 [23]	60.9 [23]	209.8	0.0065 [23]	1.36	23	1.06
Glyburide ^5^	5 [24]	0.2 [24]	0.83	0.02 [24]	0.017	11	1.00
Indomethacin ^5^	50 [28]	7.7 [28]	16.4	0.03 [13]	0.492	251	1.00
Irbesartan ^5^	300 [24]	7.7 [24]	51.2	0.1 [24]	5.12	17	1.30
Ketokonazole	200 [24]	8.5 [24]	31.9	0.01 [24]	0.319	264 [22]	1.00
Ketokonazole	200 [21]	6.6 [21]	30.0	0.01 [21]	0.3	264 [22]	1.00
Ketokonazole	200 [23]	3.2 [23]	26.6	0.01 [23]	0.266	264 [22]	1.00
Ketoprofen ^5^	100 [29]	39.7 [29]	64.2	0.03 [13]	1.92	467	1.00
Lapatinib ^5^	1250 [23]	4.2 [23]	137.8	0.01 [23]	1.38	415	1.00
Losartan ^5^	50 [24]	0.5 [24]	7.84	0.01 [24]	0.078	105	1.00
Methylprednisolone ^5^	1000 [21]	26.5 [21]	192.4	0.22 [21]	42.3	346	1.12
Nateglinide ^5^	60 [30]	15.5 [30]	27.3	0.02 [13]	0.546	290	1.00
Nefazodone ^5^	200 [31]	4.4 [31]	30.8	0.01 [13]	0.308	183	1.00
Nifedipine ^5^	10 [21]	0.23 [21]	2.02	0.0045 [21]	0.009	91	1.00
Nimodipine ^5^	30 [32]	0.11 [32]	4.56	0.02 [13]	0.091	276	1.00
Nitrendipine ^5^	20 [33]	0.12 [33]	3.56	0.02 [13]	0.071	161	1.00
Olmesartan ^5^	160 [24]	3.8 [24]	26.1	0.01 [24]	0.261	339	1.00
Pioglitazone ^5^	30 [23]	4.2 [23]	9.44	0.009 [23]	0.084	5.8	1.01
Probenecid ^5^	2000 [34]	520.8 [34]	956.1	0.12 [13]	114.7	791	1.14
Propranolol	105 [21]	0.52 [21]	25.7	0.1 [21]	2.57	6 [22]	1.43
Propranolol	80 [23]	0.19 [23]	19.4	0.13 [23]	2.51	6 [22]	1.42
Raloxifene ^5^	60 [24]	0.003 [24]	7.87	0.05 [24]	0.394	438	1.00
Rifampicin ^5^	600 [23]	7.9 [23]	53.2	0.3 [23]	16.0	605	1.00
Ritonavir	600 [21]	15.5 [21]	67.2	0.006 [21]	0.403	2 [26]	1.20
Ritonavir	600 [23]	15.3 [23]	66.9	0.015 [23]	1.0	2 [26]]	1.50
Rosiglitazone	8 [35]	1.7 [35]	3.1	0.01 [13]	0.031	5.1 [35]	1.01
Rosuvastatin ^5^	20 [36]	0.01 [36]	2.6	0.17 [37]	0.441	186	1.00
Saquinavir	400 [23]	7.6 [23]	44.6	0.02 [23]	0.892	7 [26]	1.13
Simvastatin ^5^	80 [24]	0.10 [24]	12.0	0.06 [24]	0.718	70	1.01
Sulfasalazine	1000 [35]	15.0 [35]	170.9	0.05 [24]	8.5	9.6 [35]	1.89
Telmisartan ^5^	80 [23]	1.2 [23]	10.9	0.005 [23]	0.054	87	1.00
Zafirlukast	20 [35]	0.6 [35]	2.8	0.01 [24]	0.028	6.5 [35]	1.00

^1^ Drugs are given orally except for budesonide and bulevirtide, which are inhaled and administered subcutaneously, respectively. Data for dose, I_max_, f_U,_ and IC_50_ are from the references indicated in brackets. ^2^ Calculated in this study according to Ito et al. [21] and FDA guidance [38]. I_max_: plasma concentration after a single dose; I_in,max_: estimated maximum inhibitor concentration at the inlet of the liver based on equation I_in,max_ = I_max_ + (F_a_ × F_g_ × k_a_ × Dose)/Q_h_/RB with F_a_ (fraction absorbed) = 1. F_g_ (intestinal availability) = 1. k_a_ (absorption rate constant) = 0.1/min. Q_h_ (hepatic blood flow rate) = 1616.7 mL/min. RB (blood-plasma ratio) = 1; I_in,max,u_: estimated free maximum inhibitor concentration at the inlet of the liver based on equation I_in,max,u_ = I_in,max_ × f_u_ with f_u_: fraction unbound. ^3^ Taurocholate used as substrate, except for gemfibrozil, where rosuvastatin was used. ^4^ R = (1 + ((I_in,max,u_)/IC_50_)). Drugs with a cutoff of R ≥ 1.1 are potential clinical inhibitors according to FDA guidelines [38] and are marked in gray. ^5^ IC_50_ values calculated in this study from K_i_ values based on equation IC_50_ = K_i_ × (1 + [S]/K_m_) [39] with [S] = 10 µM taurocholate and K_m_ = 22 µM [40].

**Table 2:** Correlation of DNA methylation at individual CpG sites with mRNA or protein levels of *SLC10A1*/NTCP.

	*SLC10A1* mRNA		NTCP Protein	
CpG_site	Correlation Coefficient	Unadjusted P	Correlation Coefficient	Unadjusted P
1_CpG_1	0.09	0.28	0.13	0.14
1_CpG_2	−0.01	0.87	−0.08	0.37
1_CpG_3	−0.04	0.61	−0.09	0.28
1_CpG_4	−0.02	0.82	−0.07	0.44
1_CpG_5	0.16	0.06	0.05	0.55
2_CpG_1	0.01	0.91	0.05	0.54
2_CpG_2.3	−0.01	0.89	−0.03	0.72
2_CpG_4.5	−0.17	0.05	−0.15	0.08
3_CpG_1	**0.18**	**0.04**	−0.05	0.57
3_CpG_2	−0.15	0.09	−0.14	0.12
3_CpG_3	−0.14	0.11	**−0.23**	**0.010**
3_CpG_4	−0.10	0.27	−0.07	0.42
5_CpG_2	−0.08	0.39	**−0.29**	**0.002 ^#^**
5_CpG_3.4	−0.09	0.29	**−0.17**	**0.044**
5_CpG_5	−0.15	0.07	**−0.26**	**0.002 ^#^**
5_CpG_6	−0.04	0.65	−0.02	0.79
5_CpG_7	−0.10	0.25	−0.14	0.09
5_CpG_9	−0.06	0.51	−0.15	0.08
7_CpG_1.2.3	0.02	0.82	0.14	0.11
7_CpG_5	−0.02	0.78	−0.05	0.53
7_CpG_9,10	−0.04	0.66	−0.13	0.15
7_CpG_11	0.00	0.96	−0.02	0.85
7_CpG_12	−0.01	0.95	−0.01	0.91
7_CpG_14	−0.05	0.66	0.01	0.91
7_CpG_17	−0.10	0.24	0.00	0.98

Significant associations (without correction for multiple testing) are marked in gray. ^#^ CpG sites still significant after adjustment for multiple testing using Benjamini-Hochberg procedure.

## Data Availability

Transcript profiling data will be made available in EGA archive under accession number D00010001709. Raw data files will be made available upon reasonable request.

## Supplementary Materials

The following supporting information can be downloaded at: https://www.mdpi.com/1422-0067/23/13/7468/s1.
