# Supplementary material for: Hepatic Expression of the Na+-Taurocholate Cotransporting Polypeptide Is Independent from Genetic Variation
Source: Int J Mol Sci. 2022 Jul 5;23(13):7468. doi: 10.3390/ijms23137468 (PMC9267852; doi:10.3390/ijms23137468)
Supplement: Supplementary file 1 [file ijms-23-07468-s001.zip › ijms-1789068-supplementary.pdf]

Supplementary Materials: Hepatic Expression of the Na^+-Taurocholate Cotransporting Polypeptide is Independent from Genetic Variation

Roman Tremmel, Anne T. Nies, Barbara A.C. van Eijck, Niklas Handin, Mathias Haag, Stefan Winter, Florian A. Büttner, Charlotte Kölz, Franziska Klein, Pascale Mazzola, Ute Hofmann, Kathrin Klein, Per Hoffmann, Markus M. Nöthen, Fabienne Z. Gaugaz, Per Artursson, Matthias Schwab and Elke Schaeffeler

Supplementary Tables 1-8 (page 2-14)

Supplementary Figures 1-9 (page 15-24)

Supplementary Table S1. Association of *SLC10A1* mRNA expression and NTCP protein levels with non-genetic factors in 143 liver subjects.

**Non-genetic demographic and clinical factors**[#]	**mRNA**	**Protein**
Sex (68 male vs. 75 female)	n.s.	n.s.
Age [median = 58; range 7-85 years]<br>Spearman correlation	0.03<br>r_s=-0.19	n.s.
Cholestasis (23 yes vs. 117 no)	0.03	n.s.
Medication (38 yes vs. 105 no)	n.s.	n.s.
Smoking (29 yes vs. 110 no)	0.006	0.017
Alcohol consumption (46 yes vs. 92 no)	0.002	0.005
CRP [range 0.1 – 21.1mg/ml]<br>Spearman correlation	<0.0001<br>r_s=-0.33	<0.0009<br>r_s=-0.28

Effects of non-genetic factors on the *SLC10A1* mRNA expression and NTCP protein abundance was tested either by Spearman correlation tests or Wilcoxon-Mann-Whitney tests. Cholestasis, medication, smoking and alcohol consumption were defined as previously described in Nies et al. 2009 [67].
Abbreviations: r_s = Spearman correlation coefficient; n.s., not significant (P>0.05). [#]Numbers are not summing up to 143 due to missing data.

Supplementary Table S2. Annotations and frequency distribution of genetic variants in the *SLC10A1* gene region on chromosome 14.

Position (hg19)	rs number (dbSNP)	Nucleotide	Amino acid	Localization	HWE	A/A	A/B	B/B	Missing	MAF	Method
**70266071**	rs10685904	->AC	-	promoter	1.00	117	25	1	0	9.4	N
**70266071**	rs10685904*	->C	-	promoter	0.12	135	7	1	0	3.1	N
**70265937**	rs10601222	AGA>-	-	promoter	0.21	92	42	9	0	21	M, N, S
**70265828**	rs7154439	G>A	-	promoter	0.21	92	42	9	0	21	M, N, S, G
**70265755**	rs111500198	G>A	-	promoter	1.00	139	4	0	0	1.4	M, N, G
**70265632**	rs61982106	C>T	-	promoter	1.00	109	32	2	0	12.6	M, N, S
**70265608**	rs72725750	A>T	-	promoter	1.00	109	32	2	0	12.6	M, N, S
**70265576**	rs149651811	A>G	-	promoter	1.00	139	4	0	0	1.4	M, N, S
**70265144**	rs11623504	T>G	-	promoter	1.00	109	32	2	0	12.6	I, M, N, S
**70264993**	rs59351911	T>-	-	promoter		143	0	0	0	0	M, N
**70264895**	rs990320358	G>A	-	promoter	1.00	142	1	0	0	0.3	N, S
**70264696**	rs118048231	A>G	-	promoter		143	0	0	0	0	M, N
**70263911**	rs56878770	G>C/T	-	5'UTR		143	0	0	0	0	M, N
**70263855**	rs200228184	C>T	A6A	exon 1		143	0	0	0	0	M, N
**70263683**	rs202018997	C>T	A64T	exon 1		143	0	0	0	0	M, N
**70263648**	rs4646285	C>T	T75T	exon 1	0.23	121	20	2	0	8.4	M, N, S
**70261665**	rs11622925	C>T	-	intron 1	0.23	120	20	2	1	8.5	I
**70260928**	rs76385306	A>C	-	intron 1	1	98	39	4	2	16.7	G
**70257369**	rs8020042	G>A	-	intron 1	1	109	32	2	0	12.6	G
**70253140**	rs541613204	C>G	-	intron 1	1.00	142	1	0	0	0.3	N, S
**70253139**	rs546592696	C>-	-	intron 1	1.00	142	1	0	0	0.3	N, S
**70253103**	rs770421447	CACTGTGCTAAGTGC>-	-	intron 1	1.00	142	1	0	0	0.3	N, S
**70252928**	rs61329727	C>T	K151K	exon 2		143	0	0	0	0	M, N
**70252909**	rs201339654	C>T	G158S	exon 2		143	0	0	0	0	M, N
**70252900**	rs199663299	T>A/C	I161L/V	exon 2		143	0	0	0	0	M, N
**70252878**	rs200153803	A>G	I168T	exon 2		143	0	0	0	0	M, N
**70252828**	rs200149939	G>A	R185C	exon 2	1.00	142	1	0	0	0.3	N
**70252802**	rs200579289	C>T	-	intron 2	1.00	142	1	0	0	0.3	N, S

Position (hg19)	rs number (dbSNP)	Nucleotide	Amino acid	Localization	HWE	A/A	A/B	B/B	Missing	MAF	Method
**70252701**	chr14.70252701	G>A	-	intron 2	1.00	142	1	0	0	0.3	N, S
**70250500**	rs11624523	A>G	-	intron 2	0.22	88	42	9	4	21.6	I
**70248358**	rs17556915	A>G	-	intron 2	0.80	89	49	5	0	20.6	G
**70246449**	rs11626135	G>C	-	intron 2	1	108	30	2	3	12.1	G
**70246217**	rs139537133	TCT>-	-	intron 2	1.00	109	32	2	0	12.6	N
**70246018**	rs55645214	A>G	N209N	exon 3	1.00	142	1	0	0	0.3	M, N
**70246006**	rs200746820	G>C	S213R	exon 3	1.00	142	1	0	0	0.3	N
**70245977**	rs61745930	A>G	I223T	exon 3		143	0	0	0	0	M, N
**70245923**	rs150579813	G>A	S241F	exon 3		143	0	0	0	0	M, N
**70245838**	rs186343960	A>C	-	intron 3	1.00	142	1	0	0	0.3	M, N, S
**70245407**	rs56063215	A>G	-	intron 3	0.30	109	19	2	13	8.8	N
**70245239**	rs141269120	G>A/T	R252C/S	exon 4		143	0	0	0	0	M, N
**70245193**	rs2296651	G>A	S267F	exon 4		143	0	0	0	0	M, N
**70245157**	rs72547507	A>G	I279T	exon 4		143	0	0	0	0	M, N
**70245053**	rs72547506	T>C	K314E	exon 4		143	0	0	0	0	M, N
**70244900**	rs763689587	T>G	-	intron 4	1.00	142	1	0	0	0.3	N, S
**70243149**	rs199688922	G>C	-	intron 4	1.00	142	1	0	0	0.3	M, N, S
**70242852**	rs45593332	C>T	-	3’UTR	1.00	135	8	0	0	2.8	M, N, S
**70242708**	rs150123920	A>G	-	3’UTR		143	0	0	0	0	M, N
**70241873**	rs78852170	G>A	-	3'UTR	1	136	7	0	0	2.4	G
**70240103**	rs8013586	C>T	-	downstream	1.00	107	32	2	2	12.8	I
**70238964**	rs6573908	A>C	-	downstream	0.23	87	43	10	3	22.5	I

*= rs10685904 already described as -/AC insertion, but not -/C insertion.

Abbreviations: A, reference allele; B, variant allele; 3’UTR, 3’ untranslated region; 5’UTR, 5’ untranslated region; HWE, Hardy-Weinberg P-value; genotyping and sequencing methods: G = Infinium™ Global Screening Array-v2.0, I = Illumina HAP300, M = MALDI-TOF MS, N = NGS, S = Sanger.

Supplementary Table S3. *SLC10A1* haplotypes (estimated by EM algorithm) with frequencies ≥1% using genetic variants with MAF≥5% in 143 liver samples with Caucasian ancestry and their association (R package haplo.stats::haplo.score) to *SLC10A1* mRNA (qPCR) and NTCP protein levels (LC-MS/MS proteomics).

**Haplotype**	**rs10685904**	**rs10601222**	**rs7154439**	**rs61982106**	**rs72725750**	**rs11623504**	**rs4646285**	**rs11622925**	**rs76385306**	**rs8020042**	**rs11624523**	**rs17556915**	**rs11626135**	**rs139537133**	**rs56063215**	**rs8013586**	**rs6573908**	**Frequency (EM algorithm)**	**P-value *SLC10A1* mRNA**	**P-value NTCP protein**
**1**	-	AGA	G	C	A	T	C	G	A	A	A	G	C	TCT	A	G	A	0.568	-	-
**2**	-	AGA	G	C	A	T	C	G	C	A	A	A	C	TCT	A	G	A	0.155	0.64	0.05
**3**	AC	-	A	T	T	G	C	G	A	G	G	G	G	-	G	A	C	0.096	0.77	0.98
**4**	-	-	A	C	A	T	T	A	A	A	G	G	C	TCT	A	G	C	0.085	0.80	0.50
**5**	-	AGA	G	C	A	T	C	G	A	A	A	A	C	TCT	A	G	A	0.045	0.51	0.72
**6**	-	-	A	T	T	G	C	G	A	G	G	G	G	-	G	A	C	0.031	0.77	1.00
**7**	-	AGA	G	C	A	T	C	G	C	A	A	G	C	TCT	A	G	A	0.011	0.97	0.83
**<1%**	*	*	*	*	*	*	*	*	*	*	*	*	*	*	*	*	*	0.009	0.63	0.25

Supplementary Table S4. Germline variants in the *SLC10A1* gene and their association with *SLC10A1* mRNA expression in the LIHC cohort of The Cancer Genome Atlas (TCGA) (n = 50).

Position on chr. 14	SNP	nucleotide change	allele count	MAF	p-value (additive genetic model)	p-value (dominant genetic model)	p-value (recessive genetic model)
**69778377**	.	A>C	1	0.01	-	-	-
**69778383**	.	A>G	1	0.01	-	-	-
**69778561**	rs10559679	CAG>C	2	0.02	0.86	0.86	-
**69779260**	rs61745930	A>G	1	0.01	-	-	-
**69779301**	rs55645214	A>G	1	0.01	-	-	-
**69786331**	rs375377530	GGA>G	3	0.03	0.5	0.5	-
**69796931**	rs4646285	C>T	12	0.12	0.46	0.46	-

chromosomal position based on hg38.

Supplementary Table S5: Correlation analyses of metabolite levels with *SLC10A1*/NTCP mRNA expression and protein levels. Significant associations are marked in grey.

	***SLC10A1* mRNA**				**NTCP protein**		
**Metabolites**	**r_S**	**Unadjusted P**	**Adjusted P**		**r_S**	**Unadjusted P**	**Adjusted P**
2-Ethylacryloylcarnitine or Tiglylcarnitine	0.18	**3.3E-02**	7.6E-02		-0.04	6.2E-01	7.4E-01
2-Hydroxy-butanoic acid	0.19	**2.2E-02**	5.5E-02		0.13	1.1E-01	2.4E-01
2-Phenylacetamide	0.22	**7.6E-03**	**2.9E-02**		0.18	**3.1E-02**	1.4E-01
3'5'-cyclic AMP	0.30	**2.6E-04**	**2.1E-03**		0.19	**2.1E-02**	1.1E-01
4-Hydroxy-L-proline	-0.23	**6.8E-03**	**2.7E-02**		-0.15	7.6E-02	2.1E-01
4-Pyrimidine Methanamine or 2-Aminomethylpyrimidine	0.19	**2.5E-02**	6.0E-02		0.13	1.2E-01	2.6E-01
5-Oxoproline	0.18	**3.7E-02**	8.1E-02		0.07	4.3E-01	6.1E-01
Acetylcarnitine	0.06	4.9E-01	5.7E-01		-0.05	5.8E-01	7.0E-01
Acylcarnitine 10:0	-0.10	2.2E-01	3.0E-01		-0.12	1.4E-01	2.8E-01
Acylcarnitine 10:1	0.24	**4.2E-03**	**1.8E-02**		0.14	1.0E-01	2.3E-01
Acylcarnitine 12:0	-0.02	7.8E-01	8.4E-01		-0.17	**3.9E-02**	1.6E-01
Acylcarnitine 12:1	0.10	2.6E-01	3.3E-01		0.01	9.4E-01	9.6E-01
Acylcarnitine 14:0	-0.11	1.7E-01	2.4E-01		-0.22	**8.1E-03**	8.8E-02
Acylcarnitine 14:1	-0.01	8.8E-01	9.0E-01		-0.13	1.3E-01	2.6E-01
Acylcarnitine 14:2	0.16	6.3E-02	1.2E-01		0.03	6.8E-01	7.8E-01
Acylcarnitine 16:0-OH	-0.21	**1.3E-02**	**4.0E-02**		-0.26	**1.8E-03**	5.3E-02
Acylcarnitine 16:1	-0.15	6.9E-02	1.2E-01		-0.18	**3.6E-02**	1.5E-01
Acylcarnitine 16:2	-0.02	8.1E-01	8.6E-01		-0.06	4.8E-01	6.4E-01
Acylcarnitine 18:1	-0.19	**2.2E-02**	5.5E-02		-0.16	6.1E-02	1.8E-01
Acylcarnitine 18:1-OH	-0.20	**1.7E-02**	**4.8E-02**		-0.20	**1.6E-02**	1.1E-01
Acylcarnitine 18:2	-0.08	3.7E-01	4.5E-01		-0.07	4.1E-01	5.9E-01
Acylcarnitine 3:0	0.40	**9.6E-07**	**9.4E-05**		0.16	6.5E-02	1.8E-01
Acylcarnitine 4-OH	0.16	5.2E-02	1.1E-01		0.03	6.8E-01	7.8E-01
Acylcarnitine 4:0	0.20	**2.0E-02**	5.3E-02		0.10	2.4E-01	4.2E-01
Acylcarnitine 6:0	0.32	**8.1E-05**	**1.1E-03**		0.12	1.5E-01	2.8E-01
Acylcarnitine 8:0	0.16	6.4E-02	1.2E-01		0.08	3.5E-01	5.5E-01
Acylcarnitine 8:2	0.27	**9.7E-04**	**6.3E-03**		0.21	**1.3E-02**	1.0E-01
Adenine	0.17	**4.6E-02**	9.6E-02		0.14	9.8E-02	2.3E-01
Adenosine	0.21	**1.3E-02**	**4.0E-02**		0.14	8.6E-02	2.2E-01
Alpha-Ketoglutaric acid	0.30	**2.5E-04**	**2.1E-03**		0.05	5.4E-01	6.9E-01
Betaine	0.34	**4.1E-05**	**8.0E-04**		0.30	**3.3E-04**	**2.3E-02**
Choline	0.32	**1.1E-04**	**1.2E-03**		0.20	**1.9E-02**	1.1E-01
Creatine	0.37	**5.5E-06**	**1.3E-04**		0.21	**1.2E-02**	1.0E-01
Creatinine	0.21	**1.4E-02**	**4.0E-02**		0.16	6.5E-02	1.8E-01
Cyclohexylsulfamate	-0.14	1.0E-01	1.6E-01		-0.14	1.0E-01	2.3E-01
D-Pantothenic acid	-0.13	1.3E-01	2.0E-01		-0.06	5.1E-01	6.6E-01

D/L-Alanine	0.27	**1.3E-03**	**8.1E-03**		0.16	5.3E-02	1.8E-01
Dehydroascorbic acid	0.07	4.2E-01	4.9E-01		-0.15	8.1E-02	2.1E-01
Deoxycarnitine	-0.04	6.5E-01	7.4E-01		-0.08	3.6E-01	5.5E-01
Ergothioneine	0.29	**5.5E-04**	**4.1E-03**		0.19	**2.1E-02**	1.1E-01
Glycine	0.33	**6.2E-05**	**1.0E-03**		0.25	**3.2E-03**	5.3E-02
Glycine-conjugated bile acid @3.7min	-0.26	**2.0E-03**	**1.1E-02**		-0.10	2.2E-01	3.9E-01
Glycine-conjugated bile acid @3.9min	0.01	8.7E-01	9.0E-01		0.08	3.2E-01	5.3E-01
Glycine-conjugated bile acid sulfate @5min	0.12	1.4E-01	2.1E-01		0.08	3.7E-01	5.5E-01
Glycine-conjugated bile acid sulfate @7min	-0.11	1.9E-01	2.6E-01		-0.06	5.0E-01	6.5E-01
Glycocholic acid	-0.19	**2.4E-02**	5.8E-02		0.00	9.7E-01	9.7E-01
Guanidinoacetate	0.15	6.7E-02	1.2E-01		0.02	8.2E-01	8.7E-01
Guanosine	0.09	2.6E-01	3.3E-01		0.07	4.4E-01	6.1E-01
Hexose	0.14	9.8E-02	1.6E-01		0.02	8.2E-01	8.7E-01
Hippurate	0.23	**5.1E-03**	**2.1E-02**		0.11	1.8E-01	3.3E-01
Histamine	-0.01	8.9E-01	9.0E-01		0.09	2.6E-01	4.5E-01
Hydroxyisovalerylcarnitine	0.22	**9.1E-03**	**3.2E-02**		-0.01	9.3E-01	9.6E-01
Hypotaurine	0.07	3.8E-01	4.6E-01		0.03	7.4E-01	8.3E-01
Hypoxanthine	0.00	9.9E-01	9.9E-01		0.06	4.6E-01	6.1E-01
Inosine	0.16	5.5E-02	1.1E-01		0.08	3.5E-01	5.5E-01
Isovalerylcarnitine	0.22	**8.4E-03**	**3.1E-02**		0.03	7.3E-01	8.2E-01
L-Asparagine	0.26	**1.7E-03**	**9.4E-03**		0.24	**4.3E-03**	6.1E-02
L-Carnitine	0.32	**1.3E-04**	**1.3E-03**		0.21	**1.0E-02**	1.0E-01
L-Glutamic acid	0.17	**4.5E-02**	9.6E-02		0.16	5.7E-02	1.8E-01
L-Glutamine	0.25	**2.5E-03**	**1.2E-02**		0.15	8.2E-02	2.1E-01
L-Isoleucine	0.32	**1.0E-04**	**1.2E-03**		0.22	**7.9E-03**	8.8E-02
L-Leucine	0.26	**1.6E-03**	**9.1E-03**		0.21	**1.3E-02**	1.0E-01
L-Methionine	0.13	1.3E-01	2.0E-01		0.08	3.7E-01	5.5E-01
L-Phenylalanine	0.14	8.8E-02	1.5E-01		0.14	9.6E-02	2.3E-01
L-Proline	0.13	1.2E-01	1.8E-01		0.10	2.2E-01	3.9E-01
L-Serine	-0.03	7.5E-01	8.2E-01		-0.04	6.3E-01	7.4E-01
L-Threonine	0.21	**1.2E-02**	**4.0E-02**		0.13	1.3E-01	2.6E-01
L-Tryptophan	0.12	1.5E-01	2.2E-01		0.09	2.6E-01	4.5E-01
L-Tyrosine	0.19	**2.0E-02**	5.4E-02		0.16	6.0E-02	1.8E-01
Lactate	0.03	7.1E-01	7.8E-01		-0.07	4.0E-01	5.9E-01
LysoPC 16:1	-0.12	1.7E-01	2.4E-01		-0.16	5.3E-02	1.8E-01
LysoPC 18:1	-0.14	9.7E-02	1.6E-01		-0.17	**4.2E-02**	1.6E-01
LysoPC 18:2	0.10	2.4E-01	3.2E-01		0.07	4.0E-01	5.9E-01
LysoPE 20:4	-0.02	7.9E-01	8.4E-01		-0.13	1.2E-01	2.6E-01
Mannitol	-0.03	7.1E-01	7.8E-01		-0.02	8.1E-01	8.7E-01
Methionine sulfoxide	0.07	3.8E-01	4.6E-01		-0.02	7.7E-01	8.5E-01
N-Acetyl-L-aspartic acid	-0.24	**4.2E-03**	**1.8E-02**		-0.17	**4.2E-02**	1.6E-01
N-Acetylneuraminate	-0.12	1.7E-01	2.4E-01		-0.16	5.5E-02	1.8E-01
N-Alpha-Acetyl-L-lysine	0.20	**1.5E-02**	**4.3E-02**		0.29	**4.8E-04**	**2.3E-02**

N1-Acetylspermidine	-0.02	8.4E-01	8.8E-01		0.05	5.8E-01	7.0E-01
SM 34:2	-0.10	2.5E-01	3.2E-01		-0.17	**4.3E-02**	1.6E-01
SM 36:1	0.15	6.7E-02	1.2E-01		0.07	4.4E-01	6.1E-01
SM 38:1	0.28	**7.4E-04**	**5.2E-03**		0.20	**2.0E-02**	1.1E-01
SM 42:2	-0.09	3.1E-01	3.8E-01		-0.05	5.7E-01	7.0E-01
SN-Glycero-3-phosphocholine	0.38	**2.3E-06**	**1.1E-04**		0.25	**2.3E-03**	5.3E-02
Sorbitol	-0.06	4.9E-01	5.7E-01		-0.09	2.8E-01	4.6E-01
β-Alanine	0.24	**3.7E-03**	**1.7E-02**		0.19	**2.5E-02**	1.2E-01
Succinate	0.10	2.5E-01	3.2E-01		0.01	9.0E-01	9.3E-01
Taurine	0.05	5.5E-01	6.3E-01		-0.05	5.8E-01	7.0E-01
Taurine-conjugated bile acid	-0.15	6.9E-02	1.2E-01		-0.02	8.3E-01	8.7E-01
Taurocholic acid	-0.16	6.2E-02	1.2E-01		0.00	9.7E-01	9.7E-01
Trigonellinamide	-0.15	7.3E-02	1.3E-01		-0.12	1.5E-01	2.8E-01
Trigonelline	-0.10	2.5E-01	3.2E-01		-0.06	4.5E-01	6.1E-01
Urea	0.21	**1.2E-02**	**4.0E-02**		0.19	**2.0E-02**	1.1E-01
Uridine	0.18	**3.0E-02**	7.1E-02		0.18	**2.9E-02**	1.4E-01
Valine	0.38	**4.1E-06**	**1.3E-04**		0.25	**2.8E-03**	5.3E-02
Xanthine	0.14	8.7E-02	1.5E-01		0.12	1.4E-01	2.8E-01
Xanthosine	0.17	**3.7E-02**	8.1E-02		0.05	5.6E-01	7.0E-01

Supplementary Table S6. Association of genetic variants within *SLC10A1* promoter and gene region and metabolite levels. Univariate as well as multivariate analyses including the demographic and clinical factors were performed. Only significant associations either in the univariate analysis or the multivariate analysis (unadjusted P≤0.05) are shown.

Metabolites	Genetic variant	P (univariate analysis)	Adjusted P	P (multivariate analyses)	Adjusted P
4-Hydroxy-L-proline	rs6573908	0.0196	0.1797	0.0041	0.0522
4-Hydroxy-L-proline	rs11624523	0.0302	0.1797	0.0080	0.0522
4-Hydroxy-L-proline	rs10601222	0.0327	0.1797	0.0095	0.0522
4-Hydroxy-L-proline	rs7154439	0.0327	0.1797	0.0095	0.0522
Histamine	rs4646285	0.0316	0.1871	0.0143	0.0784
Histamine	rs11624523	0.0379	0.1871	0.0127	0.0784
Histamine	rs10601222	0.0425	0.1871	0.0140	0.0784
Histamine	rs7154439	0.0425	0.1871	0.0140	0.0784
Histamine	rs11622925	0.0344	0.1871	0.0179	0.0789
Adenine	rs11624523	0.0221	0.1953	0.0116	0.1206
Adenine	rs10601222	0.0355	0.1953	0.0165	0.1206
Adenine	rs7154439	0.0355	0.1953	0.0165	0.1206
LysoPE 20:4	rs11626135	0.0430	0.1537	0.0492	0.1352
LysoPE 20:4	rs8013586	0.0465	0.1537	0.0457	0.1352
LysoPE 20:4	rs11623504	0.0489	0.1537	0.0472	0.1352
LysoPE 20:4	rs139537133	0.0489	0.1537	0.0472	0.1352
LysoPE 20:4	rs61982106	0.0489	0.1537	0.0472	0.1352
LysoPE 20:4	rs72725750	0.0489	0.1537	0.0472	0.1352
LysoPE 20:4	rs8020042	0.0489	0.1537	0.0472	0.1352
LysoPE 20:4	rs111500198	0.0832	0.2034	0.0436	0.1352
Adenine	rs6573908	0.0551	0.1953	0.0286	0.1573
Xanthosine	rs45593332	0.0051	0.1117	0.0108	0.2369
Acylcarnitine 6:0	rs11624523	0.0400	0.2796	0.0472	0.2430
Acylcarnitine 6:0	rs10601222	0.0429	0.2796	0.0394	0.2430
Acylcarnitine 6:0	rs7154439	0.0429	0.2796	0.0394	0.2430
Acylcarnitine 12:1	rs111500198	0.0109	0.2393	0.0117	0.2582
Creatinine	rs6573908	0.0179	0.1897	0.0352	0.2808
Creatinine	rs11624523	0.0300	0.1897	0.0545	0.2808
Creatinine	rs10601222	0.0345	0.1897	0.0638	0.2808
Creatinine	rs7154439	0.0345	0.1897	0.0638	0.2808
Creatinine	rs45593332	0.0942	0.2961	0.0150	0.2808
L-Methionine	rs149651811	0.0109	0.2391	0.0308	0.3335
L-Methionine	rs11624523	0.0687	0.4079	0.0450	0.3335
Adenine	rs10685904*(-/AC)	0.0435	0.1953	0.1390	0.3387

Acetylcarnitine	rs78852170	0.0211	0.4635	0.0165	0.3622
Hypoxanthine	rs78852170	0.0130	0.2856	0.0455	0.3814
Uridine	rs45593332	0.0111	0.2446	0.0174	0.3825
Deoxycarnitine	rs76385306	0.0396	0.7177	0.0178	0.3924
Betaine	rs11626135	0.0431	0.3089	0.1132	0.4287
Acylcarnitine 3:0	rs78852170	0.0253	0.3945	0.0588	0.4486
Acylcarnitine 3:0	rs11622925	0.0631	0.3945	0.0407	0.4486
L-Proline	rs6573908	0.0707	0.6411	0.0498	0.5219
Succinate	rs76385306	0.0937	0.6869	0.0239	0.5260
Acylcarnitine 8:0	rs4646285	0.0144	0.1578	0.1155	0.5307
Acylcarnitine 8:0	rs11622925	0.0146	0.1578	0.1206	0.5307
Acylcarnitine 8:0	rs111500198	0.0275	0.1578	0.0627	0.5307
Acylcarnitine 8:0	rs78852170	0.0437	0.1578	0.0679	0.5307
Acylcarnitine 8:2	rs78852170	0.0164	0.1518	0.0509	0.5422
Acylcarnitine 8:2	rs11622925	0.0275	0.1518	0.0784	0.5422
Acylcarnitine 8:2	rs4646285	0.0282	0.1518	0.0734	0.5422
LysoPC 16:1	rs111500198	0.2600	0.9114	0.0384	0.5497
Hexose	rs45593332	0.0284	0.6022	0.0510	0.5963
L-Serine	rs111500198	0.0134	0.2946	0.0284	0.6247
Acylcarnitine 10:1	rs10685904*(-/C)	0.0229	0.2049	0.0566	0.6490
Acylcarnitine 10:1	rs10601222	0.0341	0.2049	0.1452	0.6490
Acylcarnitine 10:1	rs7154439	0.0341	0.2049	0.1452	0.6490
Acylcarnitine 10:1	rs11624523	0.0373	0.2049	0.1712	0.6490
Acylcarnitine 8:2	rs10601222	0.0405	0.1518	0.2858	0.7344
Acylcarnitine 8:2	rs7154439	0.0405	0.1518	0.2858	0.7344
Acylcarnitine 8:2	rs11624523	0.0414	0.1518	0.3081	0.7344
Succinate	rs45593332	0.0324	0.6869	0.0671	0.7376
Lactate	rs111500198	0.0546	0.9960	0.0433	0.7432
Urea	rs11624523	0.0328	0.2127	0.1724	0.7773
Urea	rs6573908	0.0376	0.2127	0.1743	0.7773
Acylcarnitine 4:0	rs10685904*(-/C)	0.0305	0.5905	0.0629	0.8243
Acylcarnitine 10:0	rs111500198	0.0316	0.6962	0.0761	0.8371
Guanidinoacetate	rs111500198	0.4120	0.9587	0.0447	0.8513
SM 38:1	rs45593332	0.0452	0.8572	0.0388	0.8528
Taurocholic acid	rs111500198	0.0561	0.9745	0.0393	0.8649
Glycocholic acid	rs111500198	0.0342	0.7517	0.0719	0.8721
Acylcarnitine 18:2	rs45593332	0.0481	0.9286	0.1942	0.9212
Acylcarnitine 14:0	rs149651811	0.0415	0.9130	0.0662	0.9358
Acylcarnitine 18:1	rs45593332	0.0350	0.7700	0.1474	0.9662
Acylcarnitine 16:0-OH	rs45593332	0.0402	0.8854	0.0664	0.9675
Acylcarnitine 18:1-OH	rs45593332	0.0226	0.4978	0.1087	0.9724
SM 42:2	rs78852170	0.0292	0.6432	0.0883	0.9730

Acylcarnitine 12:0	rs149651811	0.0326	0.7182	0.0529	0.9752
SM 36:1	rs78852170	0.0279	0.6135	0.0640	0.9842
Mannitol	rs78852170	0.0186	0.4091	0.0677	0.9855

*= rs10685904 already described as -/AC insertion, but not -/C insertion

Supplementary Table S7. Surrogate peptides and their mass transitions.

Protein	Peptide sequence	Mass	DP	Q1	Q3.1	CE 1	Q3.2	CE 2	Q3.3	CE 3
**NTCP**	GIYDGDLK	880	45	440.9	710.5	17	547.2	20	355.9	19
	GIYDGDLK*	888	45	444.9	718.4	17	555.4	20	359.9	19

Abbreviations: DP, declustering potential; CE, collision energy

Supplementary Table S8. Target regions of *SLC10A1* used for library preparation (hg19).

chr	start	end	strand	gene	Exon	transcript
**chr14**	70242531	70243125	-	*SLC10A1*	5	NM_003049
**chr14**	70245029	70245266	-	*SLC10A1*	4	NM_003049
**chr14**	70245878	70246097	-	*SLC10A1*	3	NM_003049
**chr14**	70252793	70253044	-	*SLC10A1*	2	NM_003049
**chr14**	70263496	70266026	-	*SLC10A1*	1 + promoter	NM_003049

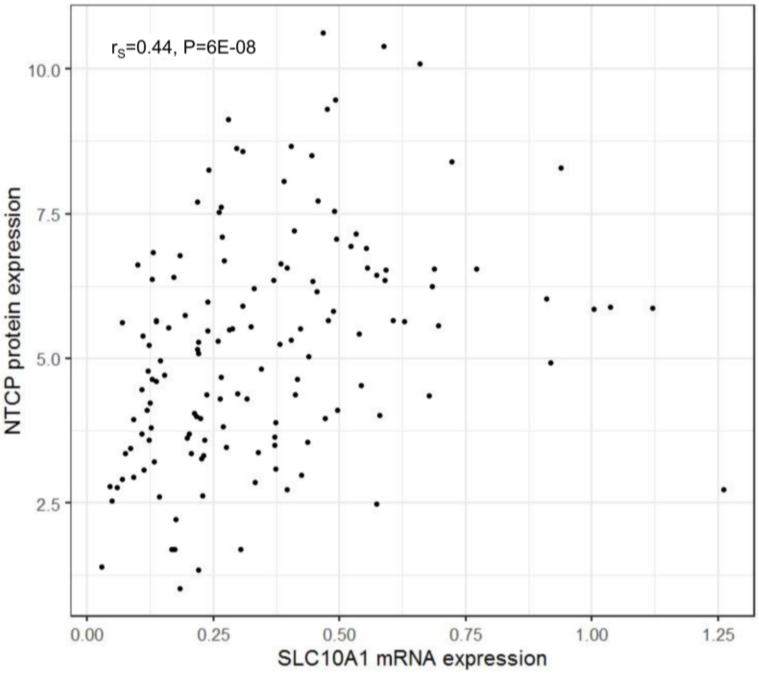

Supplementary Figure S1. Correlation analysis between *SLC10A1* mRNA expression and NTCP protein levels in liver tissue samples. mRNA expression levels were measured using qPCR and protein levels were determined using targeted LC-MS/MS. Spearman's rank correlation coefficient (r_S) and corresponding unadjusted P-value is shown.

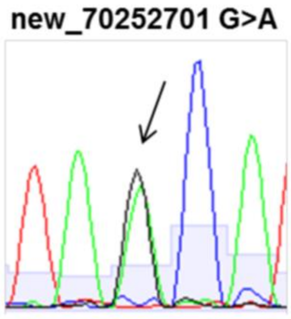

Supplementary Figure S2. Confirmation of the novel genetic variant chr14.g.70252701G>A found by next-generation-sequencing using Sanger sequencing. The figure shows the electropherogram of the respective sample with the arrow pointing to the location of the nucleotide substitution.

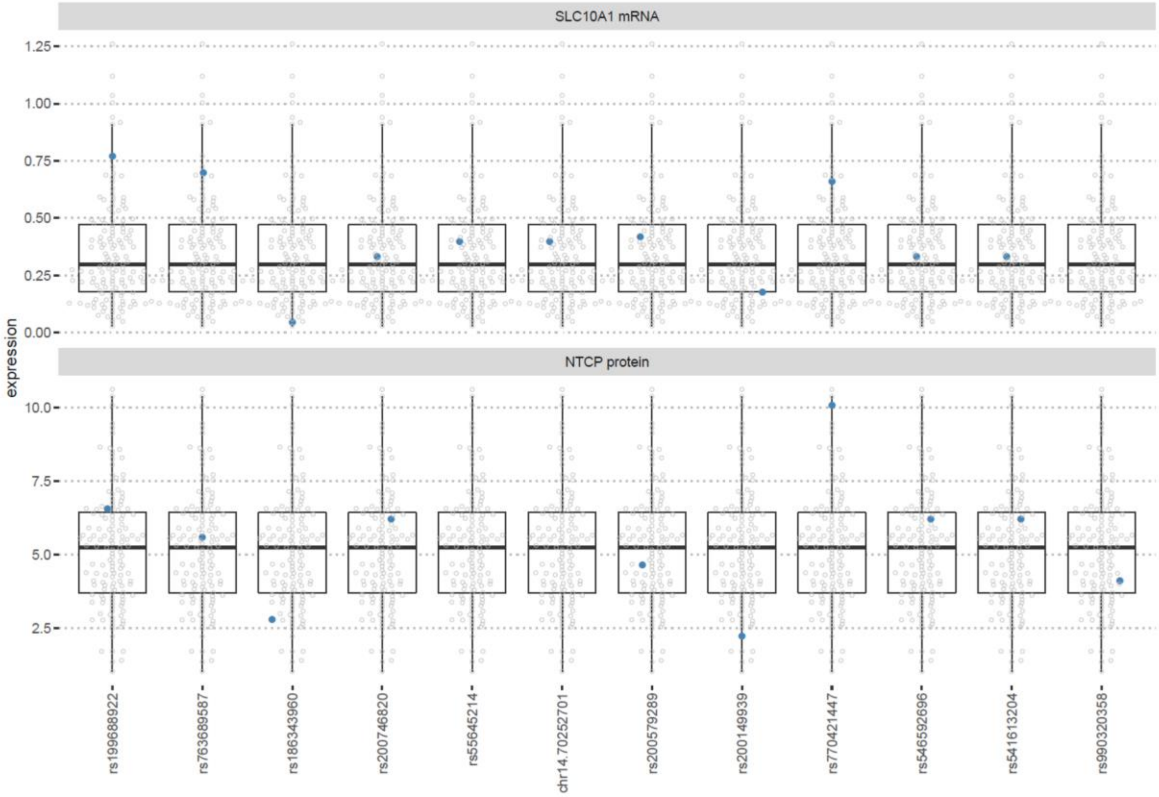

Supplementary Figure S3. Box scatter plots of *SLC10A1* mRNA expression and NTCP protein levels for 12 rare variants, including the two missense variants rs200149939 and rs200746820. Heterozygous carriers are highlighted using blue solid points, while the reference samples are displayed using grey open circles. mRNA expression was measured using qPCR while the NTCP protein levels were determined using LC–MS/MS-based targeted proteomics. Due to missing mRNA expression and protein abundance data for some subjects, the respective sample carrying the variant allele can be missing. The lower and upper hinges of the boxes correspond to the 25th and 75th percentiles. The upper and lower whisker extends from the hinge to the largest or smallest value no further than 1.5 x inter-quartile range from the hinge.

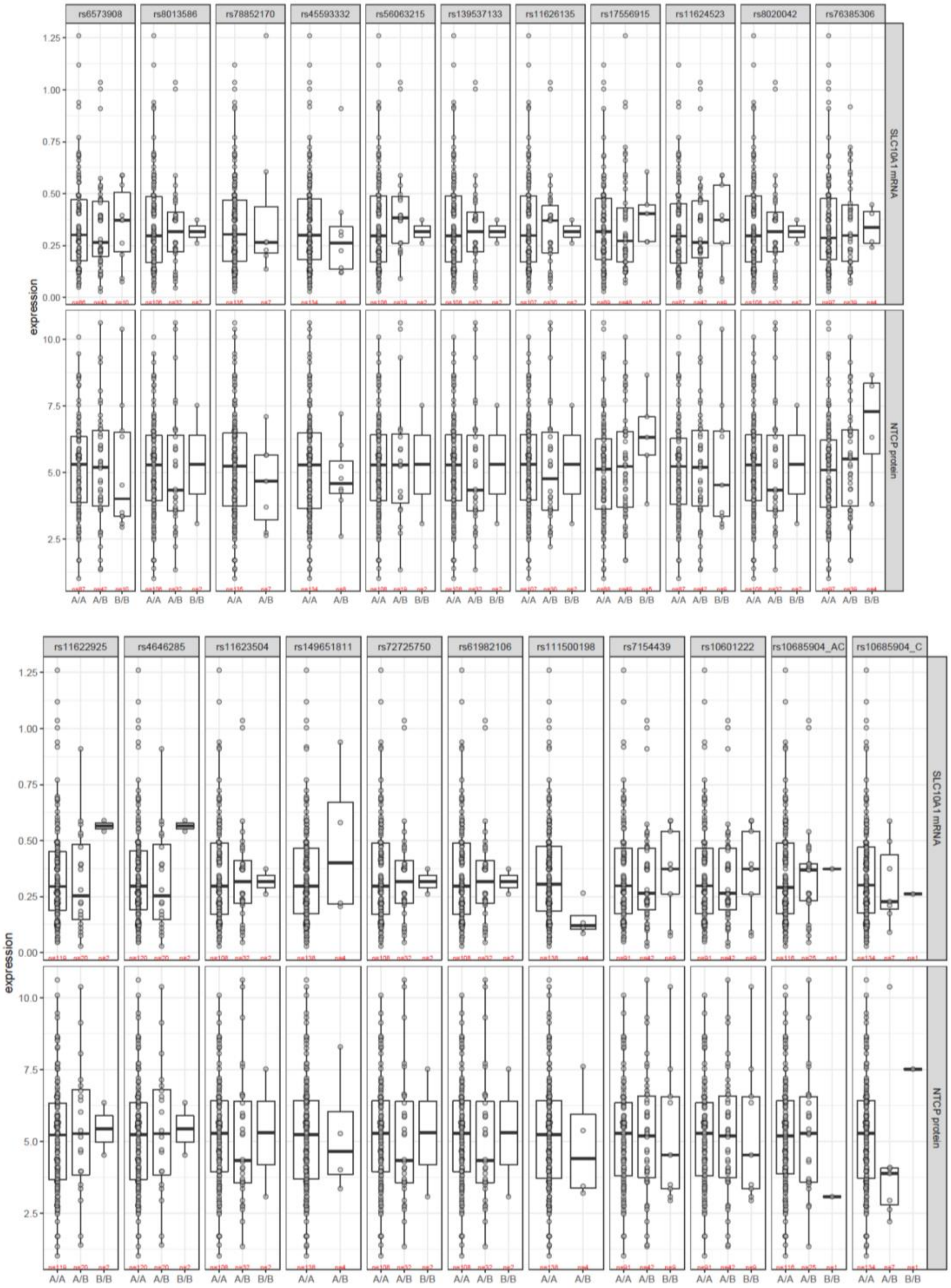

Supplementary Figure S4. Association analysis between common genetic variants and either mRNA expression or protein levels using box scatter plots. The lower and upper hinges of the boxes correspond to the 25th and 75th percentiles. The upper and lower whisker extends from the hinge to the largest or smallest value no further than 1.5 x inter-quartile range from the hinge.

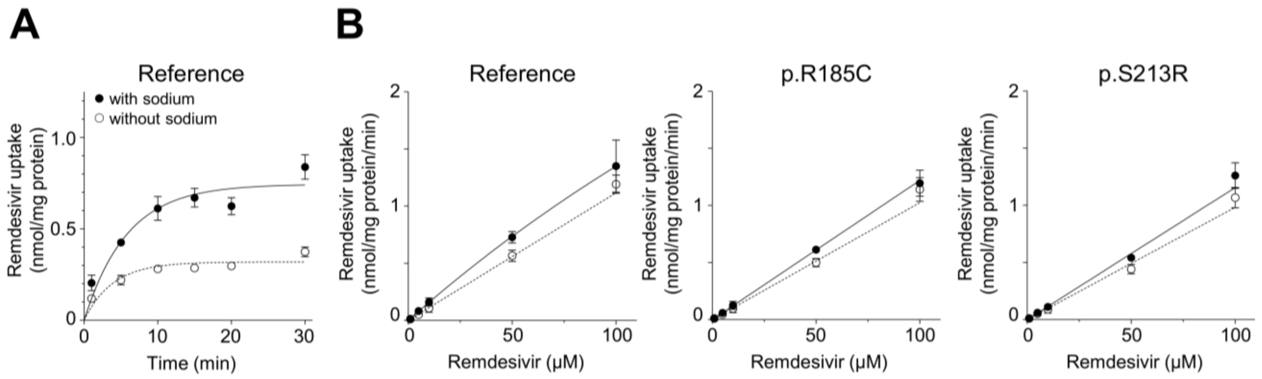

Parameter	Reference	p.R185C	p.S213R
K_m (µM)	71.0 ± 23.8	43.2 ± 12.8	219 ± 154
V_{max} (nmol/mg protein/min)	0.4 ± 0.1	0.15 ± 0.03	0.6 ± 0.4

Supplementary Figure S5. Analysis of remdesivir transport by NTCP. (**A**) Time-dependent uptake of 5 µM remdesivir into HEK cells stably expressing NTCP reference in the presence (filled circle) or absence (open circle) of sodium. Data are means ± SD of 3 wells. (**B**) Concentration-dependent uptake of remdesivir into HEK cells stably expressing NTCP reference sequence or the respective missense variant in the presence (filled circle) or absence (open circle) of sodium determined at an incubation time of 10 min. Data are means ± SD of 9 wells. Kinetic parameters were obtained by subtracting remdesivir uptake in the absence of sodium from the uptake in the presence of sodium.

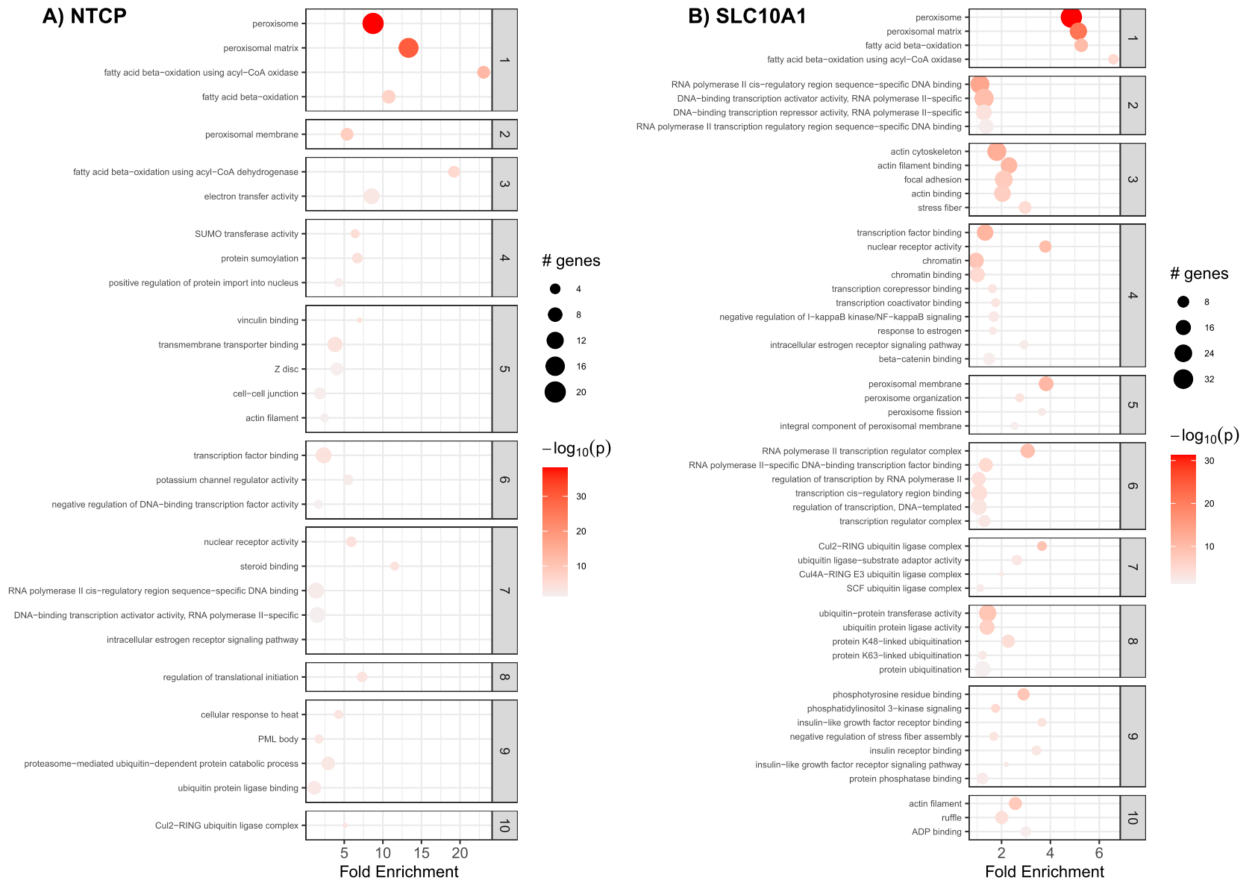
A) NTCP
peroxisome
peroxisomal matrix
fatty acid beta−oxidation using acyl−CoA oxidase
fatty acid beta−oxidation
peroxisomal membrane
fatty acid beta−oxidation using acyl−CoA dehydrogenase
electron transfer activity
SUMO transferase activity
protein sumoylation
positive regulation of protein import into nucleus
vinculin binding
transmembrane transporter binding
Z disc
cell−cell junction
actin filament
transcription factor binding
potassium channel regulator activity
negative regulation of DNA−binding transcription factor activity
nuclear receptor activity
steroid binding
RNA polymerase II cis−regulatory region sequence−specific DNA binding
DNA−binding transcription activator activity, RNA polymerase II−specific
intracellular estrogen receptor signaling pathway
regulation of translational initiation
cellular response to heat
PML body
proteasome−mediated ubiquitin−dependent protein catabolic process
ubiquitin protein ligase binding
Cul2−RING ubiquitin ligase complex
1
2
3
4
5
6
7
8
9
10
5
10
15
20
Fold Enrichment
genes
4
8
12
16
20
−log10(p)
30
20
10
B) SLC10A1
peroxisome
peroxisomal matrix
fatty acid beta−oxidation
fatty acid beta−oxidation using acyl−CoA oxidase
RNA polymerase II cis−regulatory region sequence−specific DNA binding
DNA−binding transcription activator activity, RNA polymerase II−specific
DNA−binding transcription repressor activity, RNA polymerase II−specific
RNA polymerase II transcription regulatory region sequence−specific DNA binding
actin cytoskeleton
actin filament binding
focal adhesion
actin binding
stress fiber
transcription factor binding
nuclear receptor activity
chromatin
chromatin binding
transcription corepressor binding
transcription coactivator binding
negative regulation of I−kappaB kinase/NF−kappaB signaling
response to estrogen
intracellular estrogen receptor signaling pathway
beta−catenin binding
peroxisomal membrane
peroxisome organization
peroxisome fission
integral component of peroxisomal membrane
RNA polymerase II transcription regulator complex
RNA polymerase II−specific DNA−binding transcription factor binding
regulation of transcription by RNA polymerase II
transcription cis−regulatory region binding
regulation of transcription, DNA−templated
transcription regulator complex
Cul2−RING ubiquitin ligase complex
ubiquitin ligase−substrate adaptor activity
Cul4A−RING E3 ubiquitin ligase complex
SCF ubiquitin ligase complex
ubiquitin−protein transferase activity
ubiquitin protein ligase activity
protein K48−linked ubiquitination
protein K63−linked ubiquitination
protein ubiquitination
phosphotyrosine residue binding
phosphatidylinositol 3−kinase signaling
insulin−like growth factor receptor binding
negative regulation of stress fiber assembly
insulin receptor binding
insulin−like growth factor receptor signaling pathway
protein phosphatase binding
actin filament
ruffle
ADP binding
1
2
3
4
5
6
7
8
9
10
2
4
6
Fold Enrichment
genes
8
16
24
32
−log10(p)
30
20
10

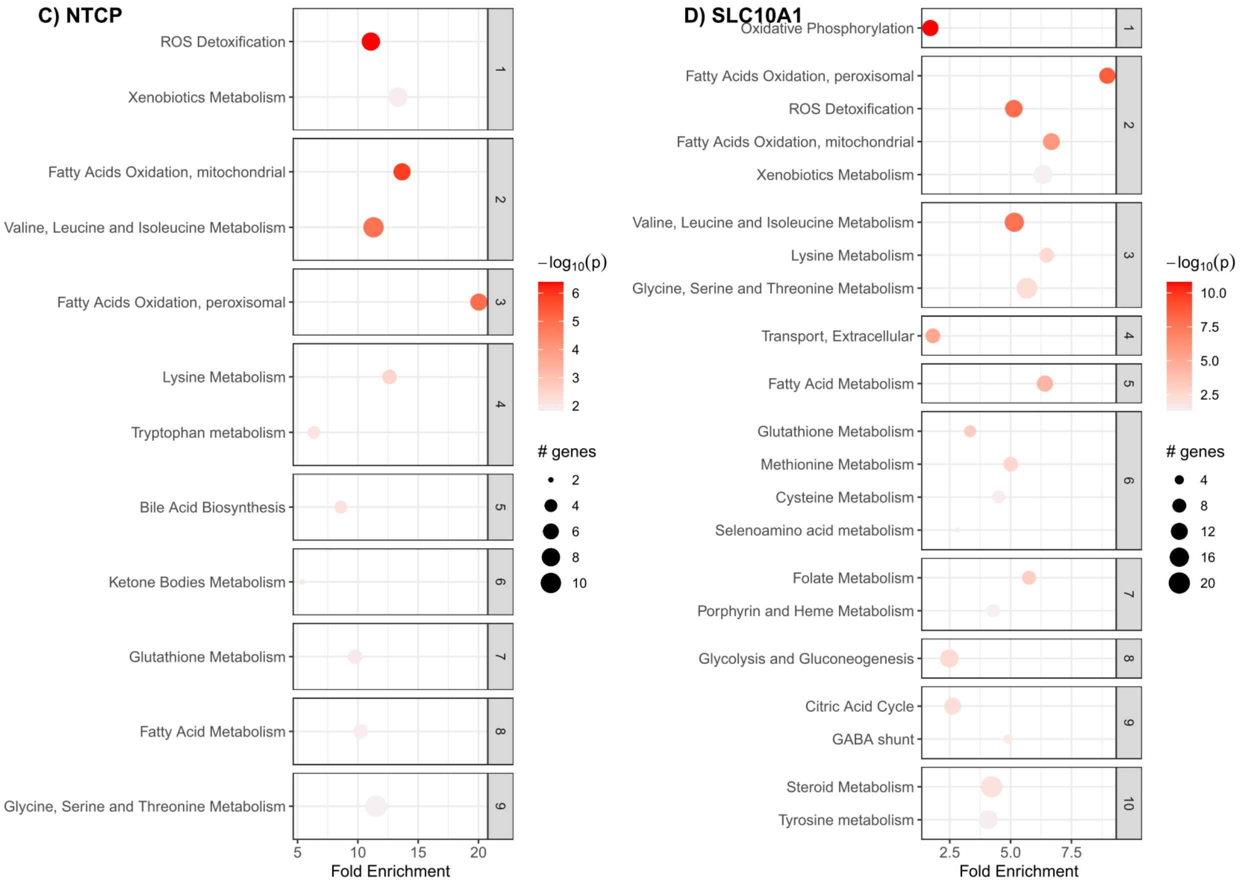

Supplementary Figure S6. Pathway enrichment results of NTCP protein and *SLC10A1* mRNA expression levels (determined by qPCR) correlated to HTA2.0 gene expression levels using GO (**A, B**) and custom gene sets derived from Gaude et al. [65]. (**C, D**) using R package pathfindR. Benjamini-Hochberg adjusted P-values were applied. The x-axis indicates fold enrichment values; the y-axis indicates the enriched pathways. The size of the bubble corresponds to the number of correlated genes in the given pathway. The orange color gradient illustrates the lowest -log10(P-values) after ten iterations. The numbers on the right side indicate the hierarchical cluster assignments.

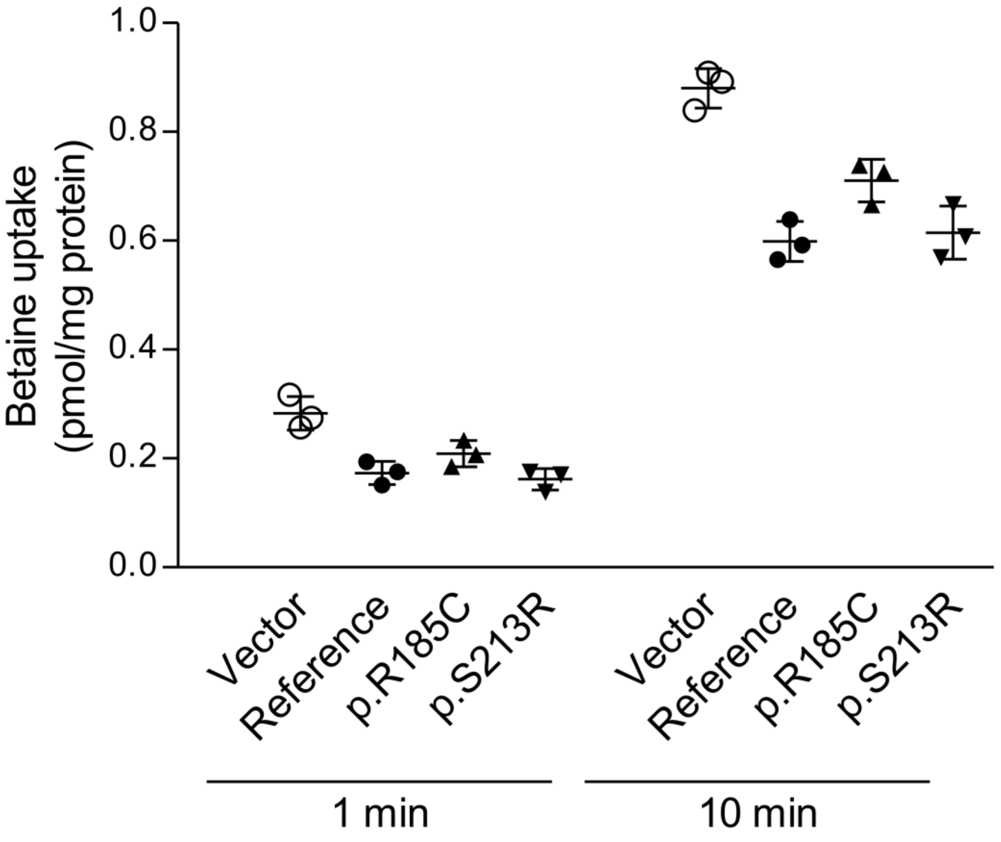

Supplementary Figure S7. Analysis of betaine transport by NTCP. Accumulation of 100 nM betaine into vector-transfected and NTCP-transfected HEK cells was measured after 1 and 10 min. Data are means ± SD of 3 wells.

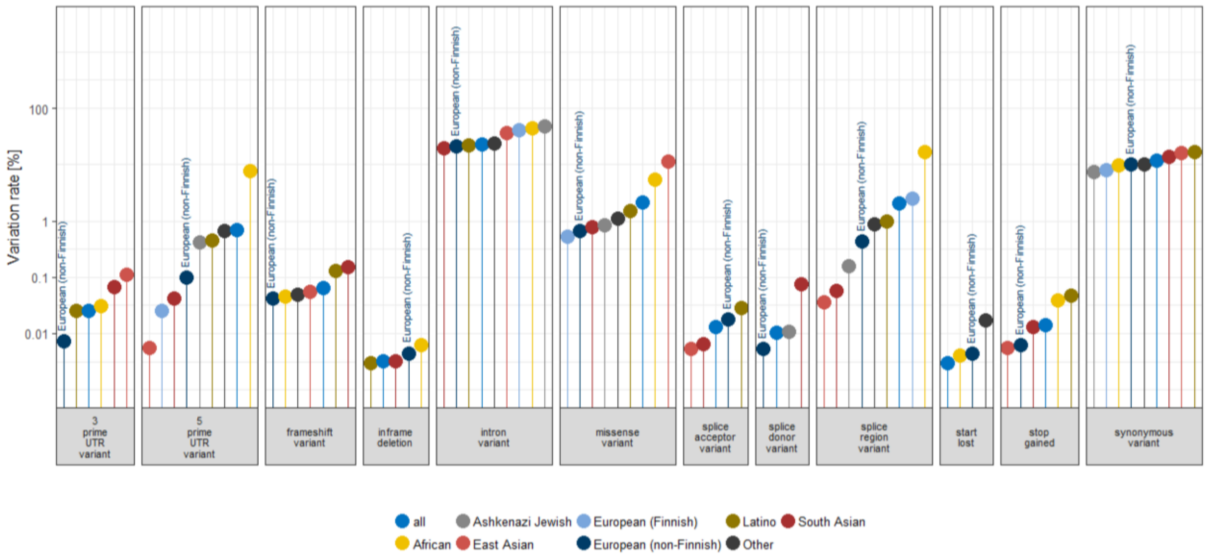

Supplementary Figure S8. Comparison of genetic variants occurring in the *SLC10A1* locus according to the gnomAD browser (gnomAD_v2.1_ENSG00000100652_2019_03_+UTR, data accessed in March 2019) stratified by population and functional annotation using variation rates. Variation rates for each population are calculated as follows: ∑MAF x npop/median(npop) where npop is the corresponding allele sample size. Of note, the median number of included samples or the allele sample size strongly differs as follows: all populations (250,778), Africans (16,254), Ashkenazi Jews (9,690), East Asians (18,390), European Finns (21,590), European non-Finns (113,420), Latinos (34,546), Others (6,135), South Asians (30,552). Variation rate [%] is given on the log10-transformed y-axis.

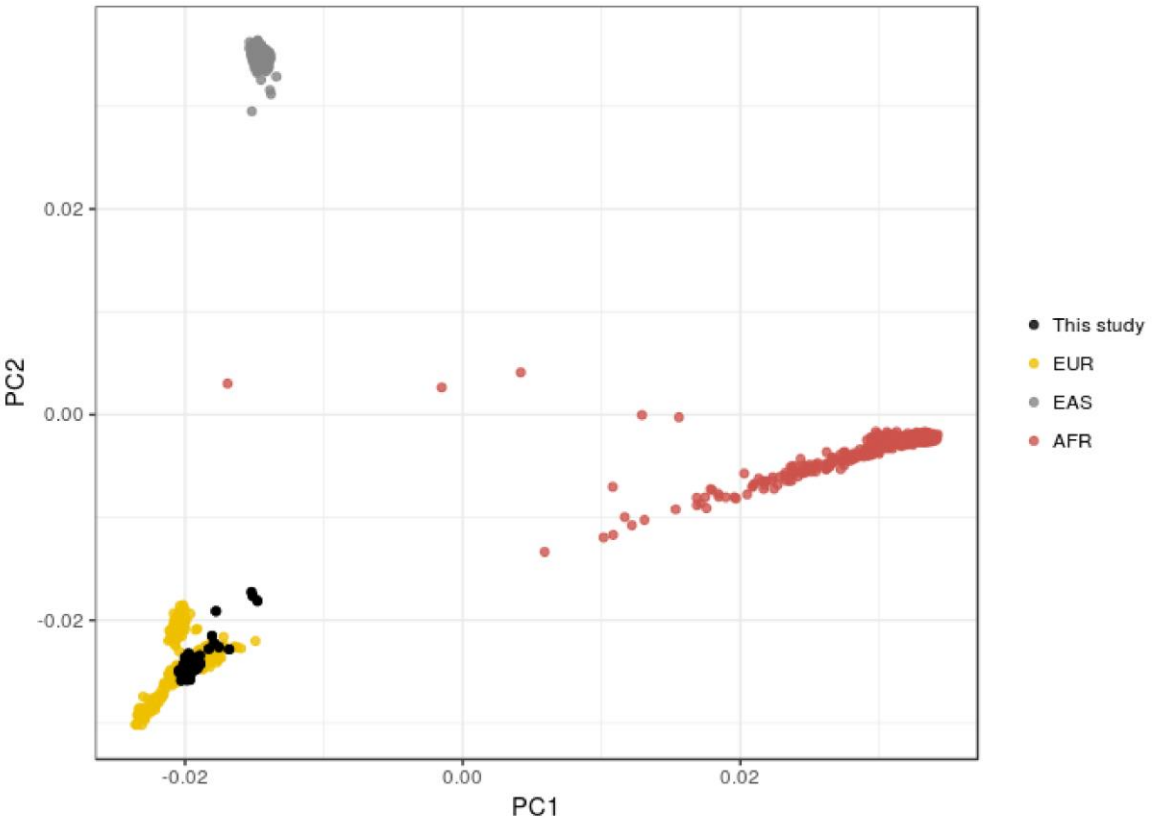

Supplementary Figure S9. Principal components analysis (PCA) of individuals from the liver cohort and subjects from different ethnicities of the 1000 Genomes project (1000G phase 3 individuals) based on 236,019 genotyped variants. PCA is based on the variance-standardized relationship matrix and was calculated using plink v1.9. The East Asian (EAS) population contains samples from CDX (93), CHB (103), CHS (105), JPT (104) and KHV (99), the African (AFR) population contains samples from ACB (96), ASW (61), ESN (99), GWD (113), LWK (99), MSL (85), YRI (108) and the European (EUR) population consists of CEU (99), FIN (99), GBR (91), IBS (107) and TSI (107). Further details on the populations and the used 1000G Phase3 data can be found at https://mathgen.stats.ox.ac.uk/impute/1000GP_Phase3.html. Individuals from the liver cohort are colored in black and 1000 Genomes AFR, EAS, EUR samples are colored in red, grey, and yellow, respectively. Cluster analysis of the 143 individuals from the liver cohort indicates that all samples were of European ancestry.
